# *In vivo* identification of apoptotic and extracellular vesicle-bound live cells using image-based deep learning

**DOI:** 10.1080/20013078.2020.1792683

**Published:** 2020-07-16

**Authors:** Jan Kranich, Nikolaos-Kosmas Chlis, Lisa Rausch, Ashretha Latha, Martina Schifferer, Tilman Kurz, Agnieszka Foltyn-Arfa Kia, Mikael Simons, Fabian J. Theis, Thomas Brocker

**Affiliations:** a Faculty of Medicine, Institute for Immunology, Munich, Germany; b Institute of Computational Biology, Neuherberg, Germany; c German Center for Neurodegenerative Diseases (DZNE), Munich, Germany; d Munich Cluster of Systems Neurology (Synergy), Munich, Germany; e Institute of Neuronal Cell Biology, Technical University of Munich, Munich, Germany; f Department of Mathematics, Technical University of Munich, Garching, Germany; g Roche Pharma Research and Early Development, Large Molecule Research, Roche Innovation Center Munich, Penzberg, Germany

**Keywords:** Extracellular Vesicles, exosomes, dendritic cells, viral Infection, irradiation, apoptosis

## Abstract

The *in vivo* detection of dead cells remains a major challenge due to technical hurdles. Here, we present a novel method, where injection of fluorescent milk fat globule-EGF factor 8 protein (MFG-E8) *in vivo* combined with imaging flow cytometry and deep learning allows the identification of dead cells based on their surface exposure of phosphatidylserine (PS) and other image parameters. A convolutional autoencoder (CAE) was trained on defined pictures and successfully used to identify apoptotic cells *in vivo*. However, unexpectedly, these analyses also revealed that the great majority of PS^+^ cells were not apoptotic, but rather live cells associated with PS^+^ extracellular vesicles (EVs). During acute viral infection apoptotic cells increased slightly, while up to 30% of lymphocytes were decorated with PS^+^ EVs of antigen-presenting cell (APC) exosomal origin. The combination of recombinant fluorescent MFG-E8 and the CAE-method will greatly facilitate analyses of cell death and EVs *in vivo*.

## Introduction

Billions of cells die every day in physiological and developmental processes [1]. Also, during viral infections cells are killed either directly by pathogens or by the immune system to limit pathogen expansion. However, despite high frequencies of cell death, it is extremely difficult to detect apoptotic cells *in vivo* [2] due to lack of appropriate detection methods and highly efficient removal of dead cells by phagocytic macrophages [3].

One hallmark of apoptotic cell death is the exposure of phosphatidylserine (PS) on the outer membrane surface of cells [4,5]. In addition to apoptotic cells, also extracellular vesicles (EVs) are PS^+^ [6–10]. EVs are very heterogeneous [11] and contain distinct nucleic acid, lipid and protein cargo derived from parental cells [12]. They may contribute to cell-to-cell communication and modulate physiological functions such as immunity, cancer progression, metastasis and transfer of viral genomes [13–15]. The concentration of EVs in bodily fluids can increase during cell death, cancer or infections [13,14]. However, the major challenge to understand the role of EVs in biological processes is to study naturally occurring EVs *in vivo* as well as their target cells. This challenge remains unsolved, as specific reagents and analysis methods are lacking.

Fluorescently labelled Annexin V, which binds to PS, has been used to detect both, PS^+^ apoptotic cells and EVs [16]. However, Annexin V requires elevated Ca^2+^-concentrations for PS-binding, which generates Ca^2+^-phosphate microprecipitates of EV-size, which can be mistaken for EVs [17]. Furthermore, the Ca^2+^-requirement might make *in vivo* applications of Annexin V difficult and could interfere with many downstream applications [18].

To reliably analyse PS^+^ EVs and dead cells *in vivo*, we have developed a recombinant PS-staining reagent by fusing Milk fat globule-EGF factor 8 protein (MFG-E8) [19] to enhanced green fluorescent protein (eGFP). MFG-E8 binds PS in a Ca^2+^-independent fashion with high sensitivity [20] and already on early apoptotic cells [21]. Furthermore, it binds to highly curved membranes [22], as those of small EVs.

Upon intravenous injection of MFG-E8-eGFP we performed imaging flow cytometry of fresh tissue cells on an ImageStream^x^ MarkII imaging cytometer, which allows detection of small particles with high sensitivity [23] and generates detailed images of individual cells [24]. To automatically classify apoptotic vs. EV-decorated (EV^+^) cells, we developed a convolutional autoencoder (CAE) [25–27], which combines the advantages of traditional feature extraction [28–30] and deep learning [31] for imaging flow cytometry. Using this pipeline, we show that MFG-E8-eGFP detects apoptotic as well as EV^+^ cells *in vivo*. In untreated mice EV^+^ haematopoietic cells are readily detectable at low frequencies *in vivo*. In contrast, irradiation or infection of mice with Lymphocytic choriomeningitis virus (LCMV) dramatically raised the frequencies of apoptotic and EV^+^ cells. Here, we analysed B cells, DCs and T cells among, which we detected a striking increase of EV^+^ cells and determined markers present on EVs to determine their origins.

We provide a novel recombinant PS-binding molecule MFG-E8-eGFP, which, in combination with the deep learning CAE tool will give valuable information on the generation and function of EVs as well as on their target-cell specificities and will be most suitable to identify cell death *in vivo.*

## Materials and methods

### Mice

C56BL/6 mice were analysed in sex and age-matched groups of 8–10 weeks of age. The SPF-status of the facility was tested according to the Federation for Laboratory Animal Science Associations (FELASA) recommendations. Animal experiment permissions were granted by the animal ethics committee of the Regierung von Oberbayern, Munich, Germany. All mice were bred and maintained at the animal facility of the Institute for Immunology, Ludwig-Maximillians-Universität München.

### Generation of recombinant MFG-E8 reporter proteins

Murine MFG-E8 full length (MFG-E8 isoform 1, NCBI Reference Sequence: NP_032620.2, AA 1-M to AA 463-C) and MFG-E8 C1C2-variant (MFG-E8 isoform 1, NCBI Reference Sequence: NP_032620.2, aminoacid position 1(M) to 22(A) and 146(S) to 463(C) were fused to eGFP (GenBank: AAB02576) or mCherry (GenBank: AST15061.1) and cloned into mammalian expression vector pcDNA3.1 (ThermoFischer). Recombinant proteins were either produced from stably transfected HEK293 cells or purchased (#2,002,100; Bioconduct, France). Cells were grown in a Labfors Bioreactor (Infors, Switzerland) in 3.5 L serum-free medium (Ex-Cell 293, Sigma) for 5 days. Cells were removed from the cell culture supernatant (SN) by centrifugation (300 g, 10 min). 0.1% Triton-X 100 was added to solubilize membrane vesicles. SN was incubated under agitation for 1 h. Debris was cleared by high-speed centrifugation (40.000 g, 90 min) and filtration (0.2 µm). MFG-E8-eGFP was then purified by FLAG affinity chromatography using 10 ml of M2-FLAG agarose beads (Sigma). Bound protein was eluted using an excess of FLAG peptide (Genscript, China) in 25 mM HEPES 2% Glycerol, 200 mM L-Arginine, 200 mM L-Glutamic acid and 150 mM NaCl, pH 7.4. The eluate was concentrated using Sartorius spin columns with a cut-off of 30kDa (Sartorius). Lastly, MFG-E8-eGFP was further purified by gel filtration on an Äkta prime system with a Superdex 200 Increase 10/300 GL column (GE Healthcare). Protein was stored in 25 mM HEPES 2% Glycerol, 200 mM L-Arginine, 200 mM L-Glutamic acid and 150 mM NaCl, pH 7.4 at −80°C. Protein yield was up to 1 mg/L of culture.

### LCMV infections

LCMV Armstrong was propagated on L929 cells. Stocks were frozen at −80°C. For quantitation of virus titres focus-forming assays using Vero cells were performed as described previously [32]. For injections, viral stocks were diluted in sterile PBS. 2 × 10^5^ p.f.u. were injected intraperitonially per mouse.

### Preparation of single cells suspensions

Single cell suspensions of spleen and thymocytes were prepared by meshing organs through a 100 µm nylon mesh. BM cells were flushed out from femur and tibia with PBS + 2%FCS using syringes. Erythrocytes were either removed by ACK (150 mM NH_4_Cl 10 mM KHCO_3_ 0.1 mM Na_2_EDTA) lysis or centrifugation through a Pancoll cushion (Pancoll, PAN Biotech). Number of live cells was determined using a CASY cell counter (OMNI Life Science).

### FACS sorting of MFG-E8^+^ splenocytes and subsequent TEM

Single cell suspensions of splenocytes from LCMV infected, MFG-E8-eGFP injected mice were prepared by meshing organs through a nylon mesh and placed in PBS + 0.5% BSA. Erythrocytes were removed by centrifugation through a Pancoll cushion (Pancoll, PAN Biotech). Number of live cells was determined using a CASY cell counter (OMNI Life Science). Cells were stained with anti-CD45 APC, LIVE/DEAD^TM^ violet (Thermo Fisher, #L34955) and anti-GFP FITC. After washing, cells were prefixed in 4% EM-grade PFA (Science Services) for 20 min before sorting. Cells were sorted on a FACSAriaIII (BD Biosciences) using a 130 µm nozzle to keep shear forces to a minimum to avoid tearing off of the EVs. Cells were sorted into PBS + 0.5% BSA and pelleted at 300 g. The cells were kept pelleted throughout all fixation, contrasting and embedding steps. Cells were fixed for 15 min in 2.5% glutaraldehyde (EM-grade, Science Services) in 0.1 M sodium cacodylate buffer (pH 7.4) (Sigma Aldrich), washed three times in 0.1 M sodium cacodylate buffer before post-fixation in reduced osmium (1% osmium tetroxide (Science Services), 0.8% potassium ferrocyanide (Sigma Aldrich) in 0.1 M sodium cacodylate buffer). After contrasting in 0.5% uranylacetate in water (Science Services), the pellet was dehydrated in an ascending ethanol series, embedded in epon (Serva) and cured for 48 h at 60°C. Ultrathin sections (50 nm) were deposited onto formvar-coated copper grids (Plano) and post-contrasted using 1% uranyl acetate in water and ultrostain (Leica). TEM images were acquired on a JEM 1400plus (JEOL) using the TEMCenter and tile scans with the ShotMeister software packages (JEOL), respectively.

### Imaging flow cytometry and data analysis

5x10^6^ cells were stained with appropriate antibodies for 20 min on ice in PBS + 2% FCS and analysed on an ImageStream^X^ MKII imaging flow cytometer (Merck). MFG-E8-eGFP^+^ cells were gated using the IDEAS software. Then TIF-images of MFG-E8-eGFP^+^ cells from each sample were exported (16-bit, raw) and analysed by the CAE algorithm. The results were stored in two separated *.pop files containing the object numbers of apoptotic and EV^+^ cells. These object numbers were re-imported into IDEAS and two separate files containing only apoptotic or EV^+^ cells were generated. Next, from each sample, three files (containing either all cells, only apoptotic or only EV^+^ cells) were exported as fcs-files which were then further analysed using FlowJo.

### Spot analysis

A spot mask was generated to identify MFG-E8^+^ ((Dilate(Peak(M02_Channel02_Bright_3)_1)_4) spots or EV-marker^+^ ((Dilate(Peak(M11_Channel11_Bright_3)_1)_4) spots. The spot count feature of the IDEAS mask was used to quantify the number of spots. To determine if EV-marker^+^ and MFG-E8^+^ spots colocalised the bright detail similarity (BDS) feature of the IDEAS software was used on the spot masks.

### Preparation of PKH26-stained EVs

40x10^6^ thymocytes were labelled with PKH26 red (Sigma Aldrich) according to the manufacturer’s protocol. Briefly, the cell suspension was washed with serum-free DMEM medium (GIBCO) and resuspended in 1 ml of dilution buffer from the manufacturer’s labelling kit. The cell suspension was mixed with an equal volume of the labelling solution in the dilution buffer and incubated for 5 min at RT. Labelling reaction was stopped by addition of 2 ml foetal bovine serum (FBS) followed by washing with complete DMEM (10% FBS, 1% Penicillin). To induce apoptosis, cells were treated with 1 μg/ml of Staurosporine (Sigma Aldrich) in serum free DMEM for 2 h at 37°c followed by three washes. Cells were removed by centrifugation (500 g). To collect PKH26-labelled vesicles, including apoptotic bodies, supernatant was ultra-centrifuged at 100,000 g for 90 min. Prior to injection into mice, vesicles were resuspended in PBS.

### Data sets used for deep learning

All datasets examined in this study were acquired using the ImageStream^X^ MKII (Luminex). For the machine learning approach only brightfield images and MFG-E8-eGFP^+^ or PKH26^+^ fluorescent images were used. All images were cropped to 32 × 32 pixels and exported as 16-bit raw TIF images. No further pre-processing was performed on the pixel intensities (e.g. normalization or scaling). The *in vitro* annotated training dataset D1 consists of 27,639 cells (27,224 apoptotic, 415 EV^+^). The apoptotic cells in this dataset were stained with MFG-E8-eGFP *in vitro*, while the 415 EV^+^ cells were prepared from splenocytes after injection of PKH26-labelled vesicles. The *in vivo* annotated dataset D2 consists of 200 cells (100 apoptotic, 100 EV^+^). The M4 *in vivo* dataset consists of 382 cells (199 apoptotic, 183 EV^+^). The M1, M2, and M3 datasets were BM cells acquired from 3 irradiated mice and consist of 14,922, 16,545 and 17,111 unannotated cells, respectively. The M5 and M6 datasets were acquired from BM of two non-irradiated mice and consist of 5805 and 5046 unannotated cells, respectively. Datasets D1 and D2 were imaged with a 40x objective, while datasets M1, M2, M3, M4, M5 and M6 were imaged with a 60x objective.

### Data analysis strategy

A novel pipeline combining unsupervised deep learning with supervised classification is used for cell classification, and compared to deep learning and classical feature-based classification.

#### Convolutional autoencoder (CAE)

The CAE used in this study consists of a typical encoder-decoder scheme but with a channel-wise adaption: the encoder part is different for each input channel, while the decoder part of the network is used only during training, not for testing. The CAE was trained on 90% of M1 for 300 epochs, while the instance of the network that performed the best on the 10% validation set of M1 was saved and used for feature extraction in all subsequent experiments. The CAE consists of approximately 200,000 parameters and the exact architecture is shown in supplementary Figure S2. Each convolutional layer is followed by a batch normalization layer [batchnorm] and a ReLU activation [relu-glorot], with the exception of the last convolutional layer which is followed by a linear (activation) function (and no batch normalization). The mean squared error (MSE) of the reconstructed image was used as a loss function for training, while the mean absolute error (MAE) produced similar results in terms of classification accuracy. Adam [adam] was used to train the network, using a batch size of 64.

#### Convolutional neural network (CNN)

The CNN used in this study for comparison is the exact same architecture as in [31] and consists of approximately 3 million parameters. For comparison to the CAE, we also implemented a smaller version of the CNN architecture where each layer of the original architecture had 1/4 of the parameters, which resulted in a model with approximately 200 thousand parameters (same as the CAE). There was no significant difference between the performance of the original and downsized variants of the CNN in any of the experiments. As such, only the results of the original variant of the CNN are reported. This specific CNN architecture receives 64 × 64 images as input, while the available images are 32 × 32. As a result, all input images were padded with their edge values to fit the input dimension of the network. In all experiments the CNN was trained using Adam [33].

#### Cell-profiler features

To compare to classical machine learning, the Cell-Profiler (CP) [29] pipeline from Blasi et al. [28] was used for feature extraction. However, in our case the second channel corresponds to fluorescence intensity instead of darkfield.

#### Random forest

The scikit-learn [34] Python implementation of the Random Forest [35] algorithm was used. The number of trees (n_estimators) was set to 1000, while the number of features to assess at each split (max_features) was set to “sqrt”. In all subsequent experiments when we refer to CAE or CP accuracy, we mean the accuracy obtained by a Random Forest trained on the pretrained CAE (CAE-RF model) features or CP features (CP-RF model), respectively.

#### CAE-RF/CP-RF

Both terms refer to a random forest trained on top of the features extracted using the pre-trained CAE introduced above or using Cell-Profiler, respectively. As such, training CAE-RF/CP-RF refers to training only the classification part of the method (RF).

#### Confidence intervals

Wilson’s method [36] was used to calculate the proportion confidence intervals for classification accuracy.

#### Code availability

The source code of this study is freely available at “https://github.com/theislab/dali”

## Results

### *MFG-E8-eGFP stains dying and PS^+^ live cells* in vivo

**Figure 1.**
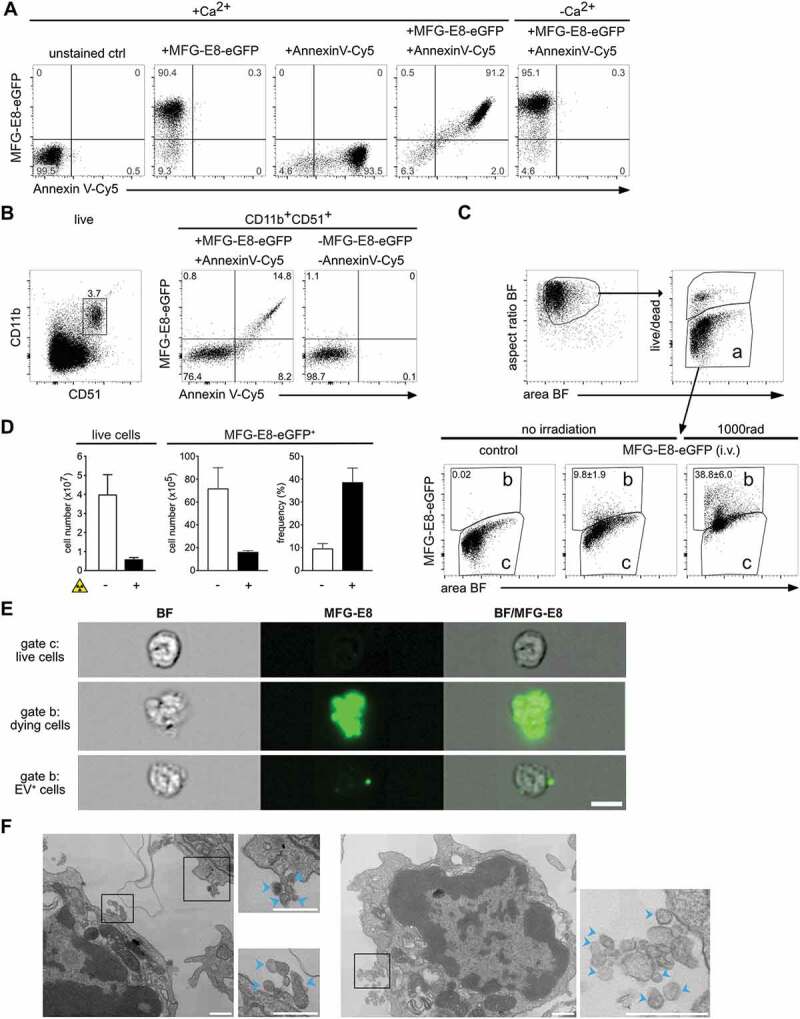
MFG-E8-eGFP stains apoptotic and EV^+^ cells *in vitro* and *in vivo*. (A) Staurosporine-treated (1 µg/ml, 2 h) apoptotic Jurkat cells were stained either with Annexin V-Cy5 or MFG-E8-eGFP or both reagents together in Ca^2+^-containing or Ca^2+^-free buffer as indicated. (B) To determine the degree of MFG-E8-eGFP binding by integrins via its RGD-motif by CD51 expressing cells, freshly isolated splenocytes were stained with MFG-E8-eGFP, AnnexinV, CD11b and CD51. Left dot plot shows gating of CD11b^+^CD51^+^ cells, middle plot shows MFG-E8-eGFP and Annexin V staining of CD11b^+^CD51^+^ cells, right plot shows unstained control. (C-D) To test MFG-E8-eGFP *in vivo* non-irradiated (n = 3) and irradiated mice (1000 rad, n = 3) were injected with 100 µg MFG-E8-eGFP i.v. 24 h after the irradiation. 30 min after the MFG-E8 injection mice were sacrificed and bone marrow (BM) cells were stained with LIVE/DEAD^TM^ violet followed by imaging flow cytometry on an Imagestream^X^ Mark II. Left bar graph shows total numbers of BM cells, middle bar and right bar graphs display the total numbers and frequencies of MFGE-E8^+^ cells with and without irradiation, respectively. Averages ± SD are shown. (E) BF and MFG-E8 images of live, MFG-E8^−^ (top), MFG-E8^+^ apoptotic (middle) and MFG-E8^+^EV^+^ (bottom) are shown. Scale bar: 7 µm. (F) MFG-E8^+^ splenocytes were FACS-sorted (sorting strategy see Supplemental Figure 2) and imaged by TEM. Two representative images of cells with attached extracellular vesicles (indicated by blue arrows) are shown. Scale bars: 500 nm.

To develop a robust, buffer-insensitive, fluorescent *in vivo* detection reagent for apoptotic cells, we fused murine MFG-E8 to enhanced green fluorescent protein (eGFP) for recombinant expression (Suppl. Fig. 1). The recombinant protein consisted of the full length MFG-E8 protein containing both C-domains (C1 and C2), of which especially the C2 domain confers PS-binding [37]. In addition, it contained the RGD-motif, which mediates binding to α_v_β_3_ integrin and facilitates phagocytosis of dead cells by macrophages [19]. The purified MFG-E8-eGFP could identify similar frequencies of dying cells *in vitro*, as compared to the commercially available Annexin V (Figure 1A). Double staining with both reagents showed that the same apoptotic cells bind Annexin V and MFG-E8-eGFP, when tested in a Ca^2+^-rich Annexin V-binding buffer (Figure 1A). However, when conventional buffer was used, only MFG-E8-eGFP, but not Annexin V could detect apoptotic cells (Figure 1A). These data indicate that MFG-E8-eGFP detects the entirety of dying cells similar to the reference reagent independently of specific buffer conditions.

The RGD-motif present in MFG-E8-eGFP can potentially also bind to α_v_β_3_ and α_v_β_5_ integrins [19]. To test if such binding might cause false-positive labelling of cells, we next stained spleen cells with anti-α_v_ (CD51) antibody, which mainly stained CD11b^+^ macrophages and monocytes (Figure 1B, left panel). However, MFG-E8-eGFP only revealed CD11b^+^CD51^+^ cells, which were also Annexin V^+^ (Figure 1B, right panel), indicating PS-specificity of MFG-E8-eGFP rather than binding via integrins.

Next, we administered MFG-E8-eGFP i.v. for *in vivo* labelling to avoid detection of artefacts generated during organ preparation, cell straining and other stress by *in vitro* handling. 30 min after injection of MFG-E8-eGFP, bone marrow (BM) cells were harvested, stained *in vitro* with a viability dye to exclude necrotic cells (Figure 1C, gate a). Due to their ruptured cell membranes, we considered necrotic cells to be too damaged to extract reliable information and we focused only on cells in the live gate (Figure 1c, gate a). Dying cells are rare in intact tissues due to their rapid removal [38]. Accordingly, only approximately 10% of all BM cells in non-irradiated mice were MFG-E8-eGFP^+^ (Figure 1c, gate b). To increase the rate of cell death, mice were γ-irradiated, which causes DNA damage and p53-mediated mechanisms of apoptosis within hours [39]. Therefore, while after irradiation the absolute numbers of cells in the BM decreased due to cell death (Figure 1d), the frequencies of MFG-E8-eGFP^+^ cells strongly increased (Figure 1c,d). This indicated the general feasibility and specificity of an MFG-E8-eGFP *in vivo* application.

The individual images taken from cells within the live populations of Figure 1c (gates a,b) showed that MFG-E8-eGFP^−^ cells had an intact rounded morphology typical for live cells (Figure 1e). In contrast, MFG-E8-eGFP^+^ cells had cell bodies that were stained almost completely with MFG-E8-eGFP and showed densely stained apoptotic blebs indicating that cells are undergoing apoptosis (Figure 1e, middle panel). However, within the same gate (Figure 1e, gate b) we also found high numbers of cells that only showed very few, or even only one intensely stained MFG-E8-eGFP^+^ structure of subcellular size, whereas their cell body was unstained and had the rounded morphology of live, intact cells (Figure 1e, lower panel). These particles were reminiscent of EVs and we next sorted MFG-E8^+^ lymphocytes from spleens for analysis by transmission electron microscopy (TEM). Attached to the sorted cells we could readily identify extracellular particles of 50–100 nm diameter, a size typical for EVs (Figure 1f). Therefore, MFG-E8-eGFP allows the analysis of apoptotic and PS^+^ EV-decorated live cells, and we next set out to characterise them in more detail.

### An interpretable deep learning approach is able to discriminate EV-decorated cells from dying cells

The imaging analysis software IDEAS is very powerful in extracting and identifying features that help to discriminate different cell subsets [40]. To generate a mask for separation of MFG-E8-eGFP^+^ cells into PS^+^ EV^+^ live or PS^+^ apoptotic cells, we manually selected 50 images of each type of MFG-E8-eGFP^+^ cells and analysed their brightfield and fluorescence characteristics (Suppl. Fig. 3A). Based on the manually selected MFG-E8-eGFP^+^ apoptotic and EV^+^ cells, we generated gates that included the majority of each cell type (Suppl. Fig. 3B). However, when we applied these definitions to cells without manual preselection in an unbiased fashion, these gates were insufficient to classify all events, leaving many cells uncategorised (Suppl. Fig. 3 C).

**Figure 2.**
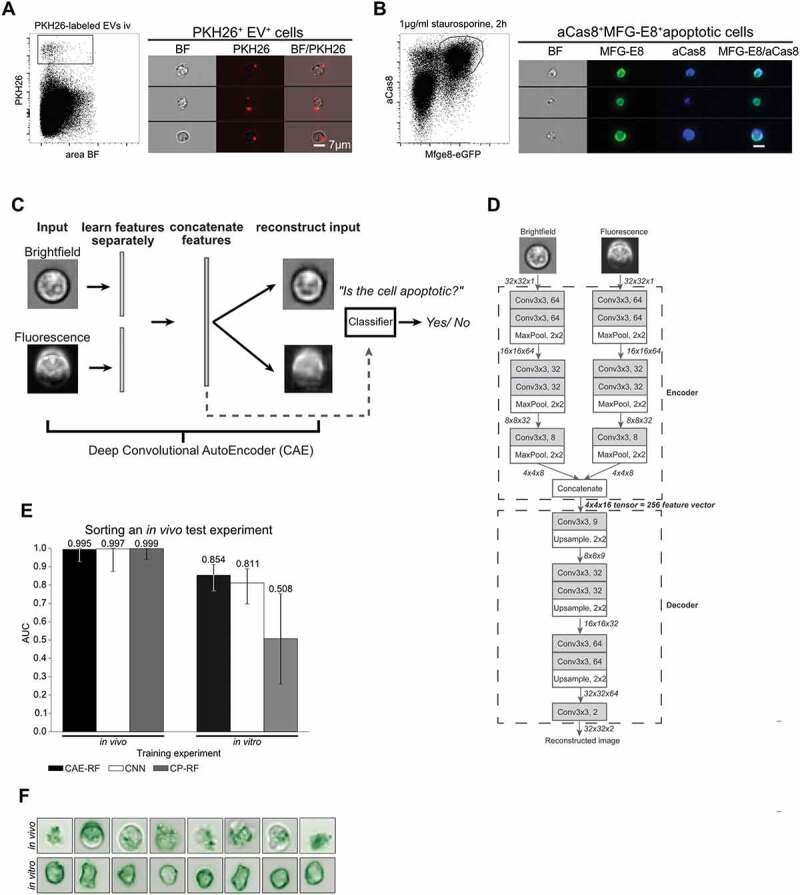
Discrimination of apoptotic vs. EV^+^ cell using machine learning. (A) To define a truth population for cells carrying EVs, PKH26-labelled EVs from staurosporine-treated thymocytes were injected i.v. into C56BL/6 mice. After 1 h spleens were removed and analysed by imaging flow cytometry. Images show splenocytes that are decorated with *in vitro* generated, PKH26-labelled EVs. (B) To define a truth population for apoptotic cells, staurosporine-treated thymocytes (1 µg/ml for 2 h) were stained with MFG-E8-eGFP (200 ng/ml) and anti-aCas8 and analysed by imaging flow cytometry. Images show MFG-E8-eGFP^+^aCas8^+^ apoptotic cells. (C) Using a Convolutional Autoencoder (CAE), both channels of the same image are separately encoded and then concatenated to form a 256-dimensional feature vector. During training features are learned in an unsupervised manner, by reconstructing the input images. In order to perform cell sorting, a classifier (Random Forest) is trained on a small subset of annotated cells. (D) Each input image consists of 32 × 32 pixels and 2 channels (Brightfield, Fluorescence). Every arrow in the figure corresponds to a data tensor. Each channel is encoded separately by an encoder network consisting of alternating convolutional and pooling layers. The encoder compresses each 32x32x1 (32x32 pixels, 1 channel) image into a 4x4x8 tensor. The encoded tensors of both channels are concatenated in a 4x4x16 tensor: the bottleneck of the CAE, which has a dual purpose. At training time, it is fed into the decoder part of the network which aims to reconstruct the input image in an unsupervised manner. At test time, the 4x4x16 bottleneck tensor is reshaped into the 256-dimensional feature vector of the input image that can be used in downstream tasks, such as classification of cell-subtypes. Each Convolutional layer performs 3 × 3 convolutions and is followed by a Batch Normalisation layer and a ReLU activation function. The only exception to this rule is the last Convolutional layer (Conv3x3, 2), which is directly followed by Linear (identity) activation function. (E) Left: All models were trained on an *in vivo* stained dataset of 401 cells (M4), then tested on an independent *in vivo* stained dataset of 200 cells (D2). Right: the same models were trained on a new dataset of 27,639 cells (D1), where the apoptotic cells were stained *in vitro*, introducing a batch effect. Next, they were tested on the same 200 cell dataset as before (D2). Sorting performance is displayed as area under curve (AUC). (F) Demonstration of the batch effect introduced by *in vitro* staining of apoptotic cells. Right column: random subset of *in vivo* stained apoptotic cells. Left column: random subset of *in vitro* stained apoptotic cells. *In vitro* stained cells fluorescent staining is evenly distributed inside the cell, while *in vivo* stained cells exhibit more complex shapes and abnormalities in the distribution of the fluorescent dye. Error-bars correspond to 95% Wilson confidence intervals (n = 200).

To identify more reliable features for apoptotic cell discrimination from EV^+^ cells, we defined a ’ground truth’ as a basis for training different classification methods for cell sorting. For this, we generated EVs *in vitro*, fluorescently labelled them with PKH26 and injected these EVs into mice (Figure 2a). Dead cells were defined using staurosporine-treated thymocytes stained with MFG-E8-eGFP in addition to the apoptotic marker active caspase-8 (aCas8) [41] *in vitro* (Figure 2b). Based on this ground truth dataset, it is possible to train a machine learning model for classification. Thus, after training is complete the machine learning method is able to separate apoptotic cells from EV^+^ cells in an automated fashion. In general, machine learning methods either operate directly on the images, or on numerical features (such as a cell’s radius and intensity) extracted from the images [42].

We next tested three different machine learning approaches with these data: (i) a Convolutional Neural Network (CNN) for imaging flow cytometry [31], (ii) our proposed method CAE-RF (a classifier trained on features learned by a CAE as displayed in the scheme of Figure 2(C,D) (iii) CP-RF, a classifier trained on pre-defined Cell Profiler features [28]. In order to estimate the effect of inter-experiment batch effects on classification performance, all methods were tested twice on the same *in vivo* stained dataset (manually selected apoptotic and EV^+^ cells from irradiated mice) and their performance was assessed using the Area Under of the receiver operating characteristic curve (AUC) [43]. The AUC metric ranges from zero to one and higher values are better since they reflect classification models with better predictive capability. An AUC score of one reflects perfect cell-sorting, while a method that randomly decides whether a cell is apoptotic or EV^+^ would achieve an AUC of 0.5. Furthermore, a batch effect can reflect slight differences in the acquired datasets, for example due to different human operators, slight differences in the stains used in independent experiments or datasets acquired by different labs. Thus, it is desirable to develop a machine learning method for cell-sorting that can overcome such batch effects.

During the first trial both, the training (MFG-E8-eGFP^+^ BM cells from irradiated mice) and test experiments were stained *in vivo*. In this case, all methods achieved near perfect classification performance of AUC>0.99 (Figure 2E). During the second trial, the apoptotic cells of the training experiment were stained *in vitro* (Figure 2B), introducing a batch effect for the classifiers to overcome (Figure 2F). In this case, CAE-RF generalised the best (Figure 2E; AUC = 0.854), followed by the CNN (Figure 2E; AUC = 0.811), while CP-RF failed to generalise to the new experiment and was comparable to random guessing (Figure 2e; AUC = 0.508). CAE-RF was as accurate as a CNN in identifying apoptotic cells in a new experiment (Figure 2e).

### For classification MFG-E8 fluorescence is more important than brightfield

**Figure 3.**
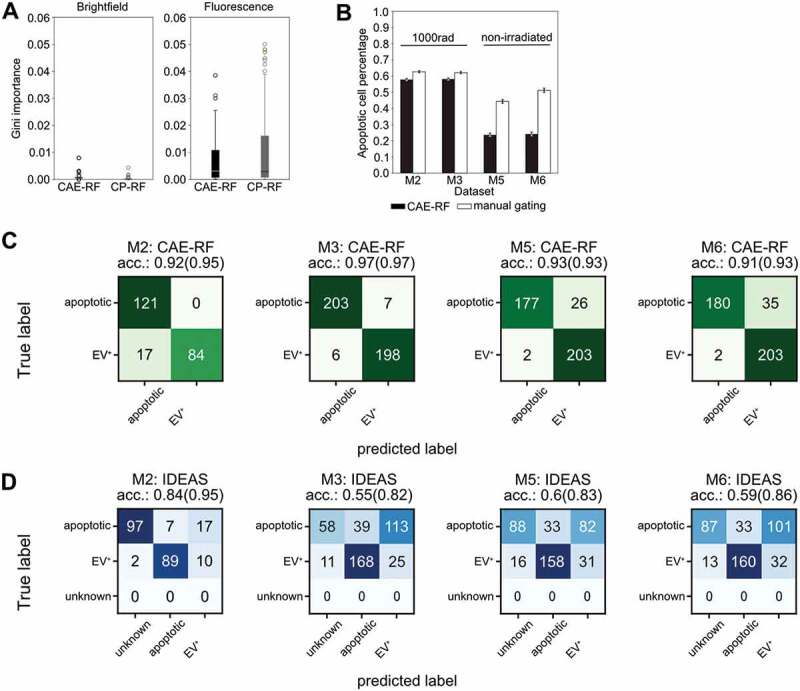
Convolutional Autoencoder performance. (a) Both CAE-RF and CP-RF identify the fluorescence channel (FL) features as more important than brightfield features (BF) for the task of apoptotic cell detection. Each boxplot visualises the Gini importance of features belonging to the corresponding channel (FL or BF), as calculated by the random forest for each feature extraction method (CAE or CP). Fluorescence features have larger values of Gini importance than BF features. (b) When predicting on new non-annotated data, both CAE-RF classification and manual gating on IDEAS features predict more apoptotic cells in irradiated mice (M2, M3) and more cells with attached vesicles in healthy mice (M5, M6). Error bars correspond to 95% Wilson confidence intervals (n_M2_ = 16,545, n_M3_ = 17,111, n_M5_ = 5805, n_M6_ = 5046). A subset of cells was annotated manually for each dataset and sorting was performed using the CAE-RF (c) and IDEAS gating (d). “Unknown” cells fail to lie on the apoptotic or EV^+^ gate using IDEAS gating. The classification accuracy reported in parentheses for each confusion matrix corresponds to the accuracy if “unknown” cells are omitted.

The CAE-RF and CP-RF methods were trained using 5-fold cross validation [43]. Both CAE-RF and CP-RF agreed that features derived from the fluorescence channel were more important than brightfield features, for the task of apoptotic cell identification (Figure 3a). Quantitative assessment of CAE-RF performance was done first by training on *in vivo* stained BM cells from irradiated mice. Then, it was used to identify apoptotic and non-apoptotic cells in new experiments with data from irradiated (Figure 3b; M2, M3) and non-irradiated mice (Figure 3b; M5, M6). The performance of CAE-RF sorting was compared to standard gating on manually defined features (Figure 3b). Both methods agree that more apoptotic cells are present in irradiated than in non-irradiated mice (Figure 3b). Subsequently, a subset of cells was manually annotated for each dataset, to quantitatively assess the classification performance of both methods CAE-RF (Figure 3c) and manual gating using IDEAS features (Figure 3d). In all cases, sorting with CAE-RF was more accurate than performing gating (Figure 3c,d). Moreover, CAE-RF always characterised all cells, while manual gating resulted in some cells characterised as ’unknown’ since they did not correspond to any of the gates (Figure 3d). Nonetheless, even if we discard the unknown cells from the calculation of classification accuracy (providing an advantage to gating), using CAE-RF was still more accurate (Figure 3d).

### True dead and live EV^+^ cells can be sorted automatically by the novel deep learning approach

**Figure 4.**
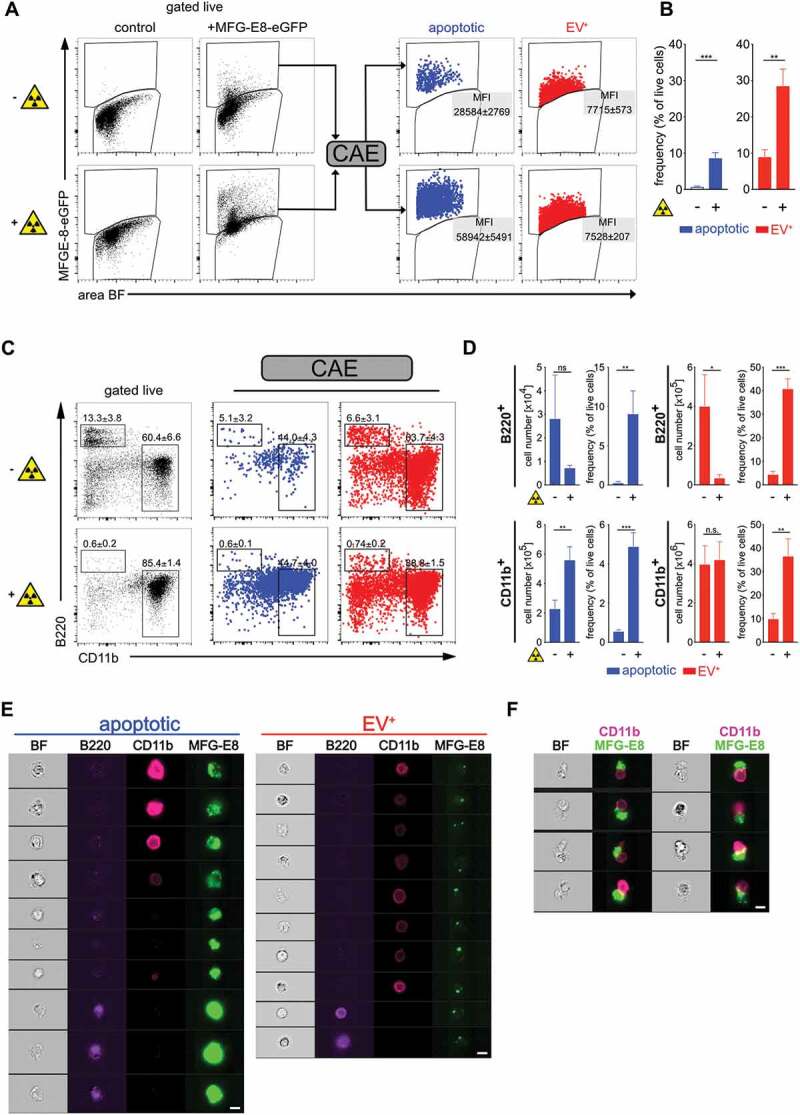
Using deep learning to discriminate apoptotic and EV^+^ cells. Non-irradiated controls (n = 3) and lethally irradiated mice (1000 rad, n = 3) were injected with 100 µg MFG-E8-eGFP i.v. 24 h after the irradiation. 30 min later mice were sacrificed. Bone marrow (BM) cells were analysed by imaging flow cytometry. (a) To identify apoptotic and EV^+^ cells, cells were analysed using IDEAS, CAE-RF and FlowJo. First, single cells were gated using the brightfield (BF) aspect ratio and the area of the BF signal. Then necrotic cells (live/dead^+^) were excluded from further analysis (Suppl. Fig. 4A). MFG-E8-eGFP^+^ cells were gated and their TIF images (16-bit, raw) exported using the IDEAS software. CAE-RF results with the classification apoptotic/EV^+^ were re-imported into IDEAS and separate fcs-files containing all cells or only MFG-E8-eGFP^+^/apoptotic cells and MFG-E8-eGFP^+^/EV^+^ cells were generated for further analysis in FlowJo. Apoptotic (blue) and EV^+^ (red) cells are shown in dot plots and their MFI of the MFG-E8 signal is displayed. (b) Bar graphs show apoptotic (blue) and EV^+^ (red) cells as frequency of live cells in non-irradiated and irradiated mice. Averages ± SD are shown. (c) Left dot plots show B220 and CD11b stained BM cells, gated on non-necrotic live/dead^−^ cells. Middle and right dot plots show B220 and CD11b expression of MFG-E8-eGFP^+^ cells classified by the CAE as apoptotic (blue) or EV^+^ (red), respectively. Numbers next to the gate show the mean percentage ± SD of all cells depicted inside the dot plot that lie within the respective gate. (D) Bar graphs show total numbers and frequencies of B220^+^ and CD11b^+^ apoptotic (blue) and EV^+^ (red) cells in non-irradiated and irradiated mice. Bar graphs show means ± SD, n = 3. (e) Example images of cells that are classified as apoptotic (left) or EV^±^ (right) are shown. (f) Example images of cells that are classified as apoptotic and are attached to CD11b^+^cells in irradiated mice. Scale bar 7 µm. Representative results of 3 independent experiments are shown. Statistical significance is indicated by asterisks (*P ≤ 0.05; **P ≤ 0.01; ***P ≤ 0.001; two-tailed unpaired t-test).

To challenge the accuracy of the CAE classifier, we next submitted image information from BM cells after irradiation to CAE-RF based sorting. As expected, the frequencies of MFG-E8-eGFP^+^ apoptotic cells strongly increased upon irradiation (Figure 4a,b). Here, only cells were included that were live/dead^−^ apoptotic cells with intact cell membranes. MFG-E8-eGFP^+^ dying apoptotic cells had a higher MFG-E8-eGFP median fluorescence intensity (MFI) than EV^+^ cells and both types could be clearly separated from each other by using CAE-RF (Figure 4a). Although the rates of both, apoptotic as well as live EV^+^ cells increased upon irradiation, the EV^+^ cells were by far more frequent than dying cells (10x more in steady-state, 3x more after irradiation, Figure 4b). We analysed both cell types further and used antibodies to CD11b^+^ myeloid cells and B220^+^ B lymphocytes, as prominent representatives of BM populations (Figure 4c). As expected, we could detect only few B220^+^ B cells in irradiated mice (Figure 4c), but a high frequency of them was classified as apoptotic (Figure 4d, upper panel). This reflected the high sensitivity of B cells to γ-irradiation [44]. In contrast, total numbers of myeloid CD11b^+^ cells were less decreased, as they have lower sensitivity to γ-irradiation [45] (Figure 4d, lower panel). However, also in the CD11b^+^ myeloid compartment, the frequencies of dying cells increased significantly upon irradiation (Figure 4c,d). Frequencies of EV^+^ live cells also increased significantly in both, CD11b^+^ and B220^+^ cells during irradiation (Figure 4c,d). Randomly selected images illustrate the different MFG-E8 staining patterns of apoptotic and EV^+^ cells (Figure 4e). Strikingly, in irradiated mice, we frequently detected apoptotic cells that were attached to CD11b^+^ phagocytes (Figure 4f). Hence, the CAE classification also correctly detects dying cells that are most likely in the process of being phagocytosed. Taken together, the combination of *in vivo* applied MFG-E8-eGFP, imaging cytometry and CAE-RF module analysis is able to reliably separate EV^+^ cells from PS^+^ dying cells in the live cell gate and can assist in more precise analyses of cell death and EVs *in vivo*.

### Distinction of dying from EV-decorated cells during acute infection with LCMV

**Figure 5.**
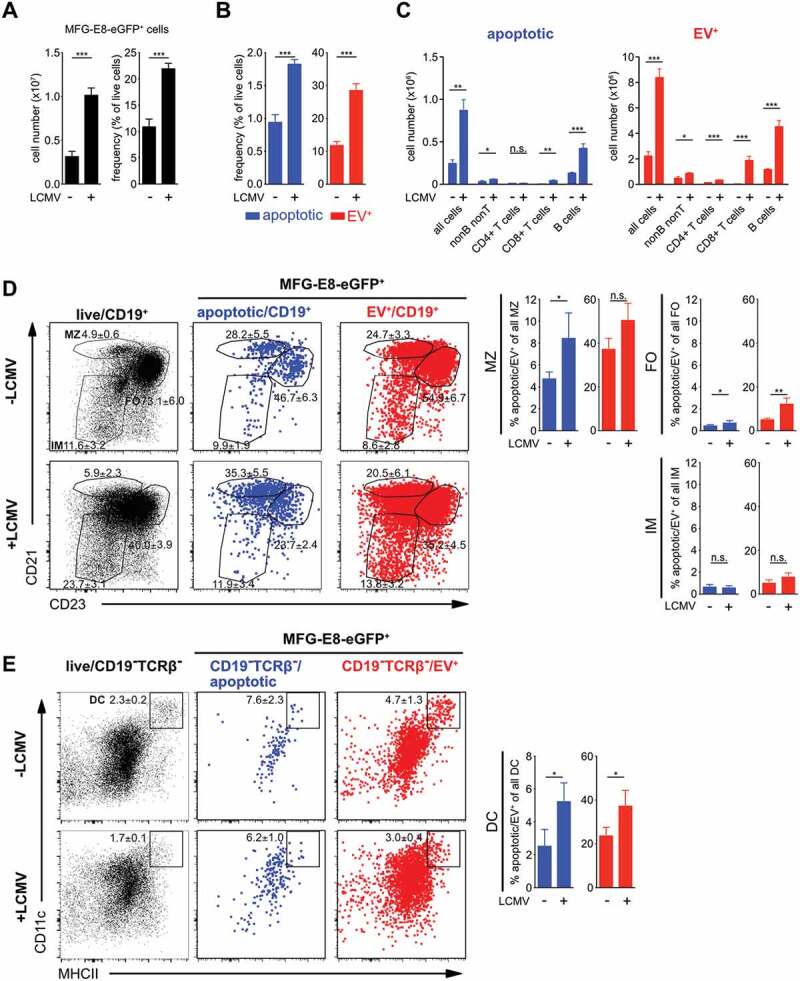
Identification of dying cells and EV^+^ cells during LCMV infection. Non-infected and LCMV_Arm_ (2x10^5^ PFU, i.p.) infected mice were injected with 100 µg MFG-E8-eGFP on day 5 post infection. 1 h later mice were sacrificed and splenic B, T and non-B/T cell subsets were analysed by imaging flow cytometry (gating strategy shown in suppl. Figure 4). (A) Bar graphs show total numbers (left) and frequencies (right) of all MFG-E8-eGFP^+^ splenocytes in non-infected and infected mice. (B) Frequencies of MFG-E8-eGFP^+^ apoptotic (blue) and EV^+^ (red) cells were determined using the CAE. (C) MFG-E8-eGFP^+^ CD19^−^TCRb^−^ nonB/T cells, CD4^+^ and CD8^+^ T cells and CD19^+^ B cells were classified as apoptotic (blue) or EV^+^ (red) using the CAE and their total numbers in the spleen were calculated. (D) MFG-E-eGFP^+^ CD19^+^ B cell subsets (MZ = marginal zone, FO = follicular, IM = immature B cells), (B) CD19^−^TCR ^−^CD11 c^+^MHC-II^+^ DCs were analysed using the CAE. Numbers next to the gate show the mean percentage ± SD of all cells depicted inside the dot plot that lie within the respective gate, while the bar graphs show the average frequency ± SD of apoptotic or EV^+^ cells within the analysed subpopulation (n = 3). Statistical significance is indicated by asterisks (ns *P* > 0.5; *P ≤ 0.05; **P ≤ 0.01; ***P ≤ 0.001; two-tailed unpaired t-test). Representative results of 3 independent experiments are shown.

Having developed a reliable automated method to discriminate apoptotic from EV^+^ cells, we next analysed spleens of mice during an LCMV infection, which is known to induce cell death during the acute infection phase [46]. Here, cell death is mainly caused by innate and adaptive immune mechanisms [46–48]. LCMV infection caused a strong increase in frequencies and total numbers of live MFG-E8-eGFP^+^ cells (Figure 5a). To differentiate apoptotic from EV^+^ live cells we CAE-sorted their images. This revealed that both, apoptotic as well as EV^+^ cells significantly increased upon LCMV infection (Figure 5b). However, the frequencies of EV^+^ live cells were more than 10-fold higher as compared to those of apoptotic cells, before, as well as during LCMV infection (Figure 5b). The paucity of apoptotic cells is striking, but also expected. In healthy mice, we detected approx. 250,000 apoptotic cells per spleen. This number increased to approx. 1 Mio. in infected mice. This low number highlights the importance of a reliable and specific method to identify dying cells. More detailed analyses showed that the highest numbers of dying cells in non-infected mice were present within the CD19^+^ B cell and CD19^−^TCRβ^−^ non-B/T cell populations (Figure 5c). Upon LCMV infection, both, dying and live EV^+^CD19^+^ B cells and CD19^−^TCRβ^−^ non-B/T cells further increased, but live EV^+^ B cells outnumbered dying B cells approximately 10-fold (Figure 5c). In addition, especially CD8^+^ T cells showed increased frequencies of apoptosis and EV-decoration upon LCMV infection (Figure 5c). Among CD19^+^ B cells, mainly marginal zone (MZ, CD19^+^CD21^hi^CD23^lo^) and follicular (CD19^+^CD21^+^CD23^hi^) B cells showed increased apoptosis, while only follicular B cells also showed a significant increase in EV^+^ cells during LCMV-infection (Figure 5d). MZ B cells showed a very high degree of EV-decoration in both infected and non-infected animals (Figure 5d). Among the CD19^−^TCRβ^−^ non-B/T cell populations CD11c^+^MHC-II^+^ DCs showed slight but significant increases of both, apoptotic and EV^+^ cells (Figure 5e).

### CD8^+^ T effector cells preferentially become EV-decorated

**Figure 6.**
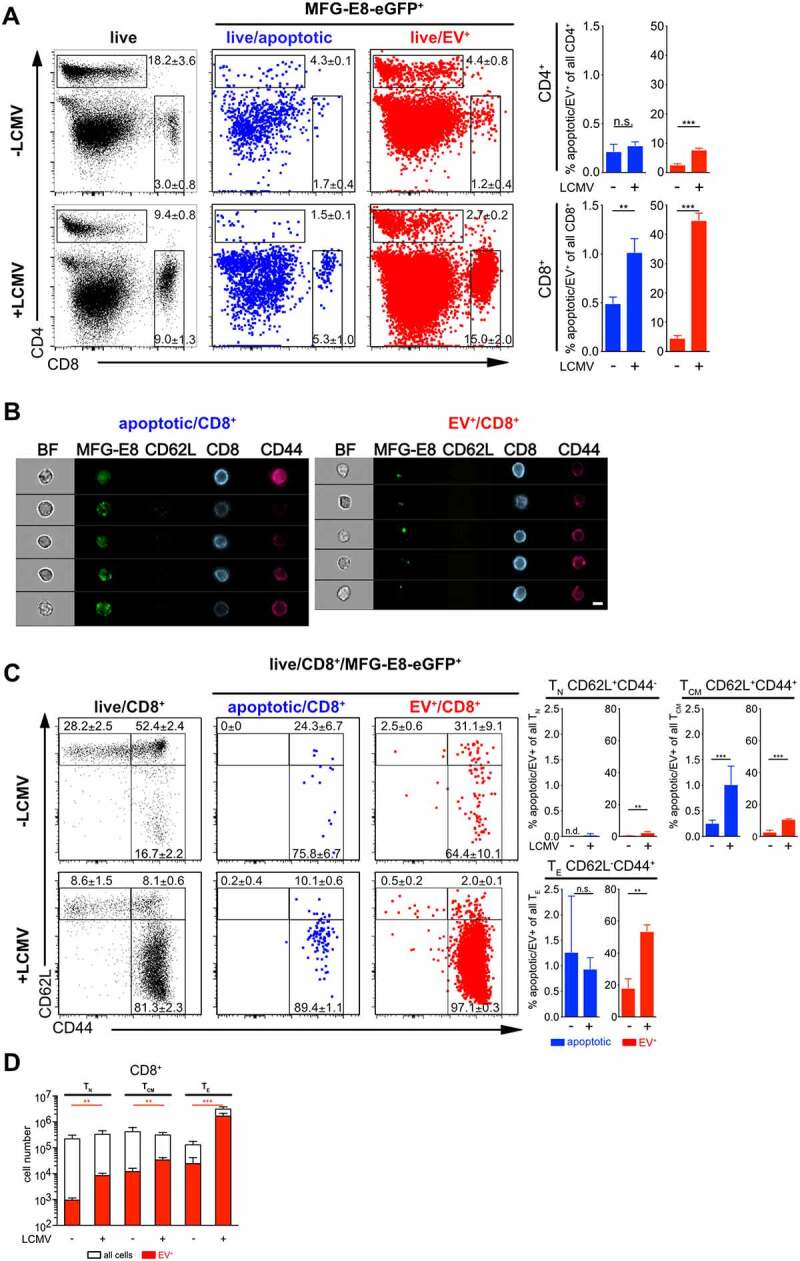
CAE analysis of T cells during LCMV infection. Non-infected and LCMV_Arm_ (2x10^5^ PFU, i.p.) infected mice were injected with 100 µg MFG-E8-eGFP on day 5 post infection. 1 h later mice were sacrificed and (A) CD4^+^ and CD8^+^ T cells and (C) CD62L^+^CD44^−^ naïve (T_N_), CD62L^+^CD44^+^ central memory (T_CM_) and CD62L^−^CD44^+^ effector memory T_E_ CD8^+^ T cells were analysed using the CAE and the percentages of apoptotic and EV^+^ cells determined. Numbers next to the gates show the mean percentage ± SD of cells that lie within the respective gate. Bar graphs show the average percentage ± SD of apoptotic or EV^+^ CD4^+^ and CD8^+^ T cells within the analysed subpopulation. (B) Representative imaging flow cytometry images of apoptotic (left) and EV^+^ (right) CD8^+^ T cells are shown. Scale bar 7 µm. (D) Total numbers of CD8^+^ T_N_, T_CM_ and T_E_ cells were determined (white bar) and plotted against the total numbers of EV^+^ subsets (red bar). Representative results of three independent experiments are shown. Bar graphs show the average frequency ± SD of apoptotic or EV^+^ cells within the analysed subpopulation (n = 3). Statistical significance is indicated by asterisks (ns *P* > 0.5; *P ≤ 0.05; **P ≤ 0.01; ***P ≤ 0.001; two-tailed unpaired t-test).

CD4^+^ and CD8^+^ T lymphocytes play central roles in controlling viral infections [49,50]. Analysis of spleens from LCMV infected mice showed that the frequencies of apoptotic cells increased during viral infection only among CD8^+^ T cells, but not CD4^+^ T cells (Figure 6a). While EV-decoration increased only slightly, but with statistical significance also in CD4^+^ T cells, it augmented drastically in CD8^+^ T cells during LCMV infection, when 40–50% of all CD8^+^ T cells were EV^+^ (Figure 6a). Flow cytometry images confirmed the morphological differences of the MFG-E8-eGFP staining between apoptotic and EV^+^ CD8^+^ T cells (Figure 6b). The strong EV-association with CD8^+^ T cells was very striking and warranted further investigation. We thus assessed which CD8^+^ T cell subsets become EV-decorated. In lymphoid organs of mice functionally homologous populations are defined as naïve (T_N_, CD62L^hi^CD44^lo^), central memory (T_CM_, CD62L^hi^CD44^hi^) and terminally differentiated effector memory cells (T_E_, CD62L^lo^CD44^hi^) [51]. In steady state, the frequencies of apoptotic and EV^+^ cells were extremely low and mostly found within antigen-experienced CD44^hi^ cells, but not T_N_ cells (Figure 6c). Upon LCMV-infection, CD62L^lo^CD44^hi^ T_E_ cells expanded strongly without showing an increased rate of cell death as compared to uninfected mice (Figure 6c). Only CD62L^hi^CD44^hi^ T_CM_ cells significantly increased their rate of apoptosis (Figure 6c). The reduction of total T_CM_ cell numbers confirmed this finding (Figure 6d) and has been described earlier as type I interferon depend attrition of memory T cells [52–54]. In contrast to the relatively low numbers of *bona fide* apoptotic CD8^+^ T cells, EV-decoration of CD8^+^ T cells increased significantly in all populations, T_N_, T_CM_ and T_E_ cells upon infection by frequency (Figure 6c) and cell number (Figure 6D). The highest EV-decoration frequencies were detected in CD44^hi^ T_CM_ and T_E_, the latter reached around 50% EV-decoration and more (Figure 6c). Likewise, the frequencies of apoptotic CD4^+^ T cells were generally low in non-infected mice, where only around 2% CD62L^+^CD44^+^CD4^+^ T_CM_ cells and 10% antigen-experienced CD62L^−^CD44^+^CD4^+^ T_E_ cells were EV^+^ (Suppl. Fig. 5B). Upon LCMV infection EV-decoration augmented in all CD4^+^ T cell subsets, the strongest increase was observed in antigen-experienced CD44^hi^CD4^+^ T_CM_ and T_E_ cells (Suppl. Fig. 5A, B). Also in the CD4 compartment, total CD4^+^ T_CM_ cell numbers declined and the frequencies of apoptotic cells increased, suggesting that CD4^+^ T_CM_ were eliminated by apoptosis in the early phase of infection (Suppl. Fig. 5A, B, C). Analysis of total cell numbers of EV^+^ T cells revealed significant increases in EV^+^ cells in all CD4^+^ (Suppl. Fig. 5 C) and CD8^+^ (Figure 6c) T cell subsets upon LCMV infection. However, the strongest increase was found in CD8^+^ T_E_ cells, which exceeded all other T cell subsets in terms of EV-decoration (Figure 6c, Suppl. Fig. 5 C), indicating a high disposition of CD8^+^ T cells to associate with EVs.

### EV-decoration of activated CD8^+^ T cells is not mediated through RGD-motif

To address the possibility that the RGD-motif present in full-length MFG-E8-eGFP mediates attachment of EVs to activated T cells, we assessed binding of EVs stained with an MFG-E8 variant lacking the PT, E1 and E2 domains, the latter of which contains the integrin-binding RGD-motif (C1C2-eGFP, Suppl. Fig. 6A). Upon i.v. injection of equimolar amounts of full-length MFG-E8-eGFP or C1C2-eGFP similar amounts of eGFP^+^ cells were detected (Suppl. Fig. 6B), although the C1C2-variant of MFG-E8 was reported to have decreased affinity to PS as compared to full length MFG-E8 [19]. Both MFG-E8 versions detected the same frequencies of apoptotic and EV^+^ cells (Suppl. Fig. 6B). Also, the ability of CD8^+^ T_E_ to bind EVs was not affected by the absence of the RGD-motif, as a similar frequency to EV^+^CD44^+^CD8^+^ T_E_ were detected, regardless of the presence of the RGD-motif in the MFG-E8 construct (Suppl. Fig. 6 C). Therefore, MFG-E8, with or without RGD-motiv, binds to T cells already associated with EVs rather than inducing this association.

### In vivo *MFG-E8 staining mirrors PS^+^ EVs and PS^+^ apoptotic cells more reliably than* in vitro staining

**Figure 7.**
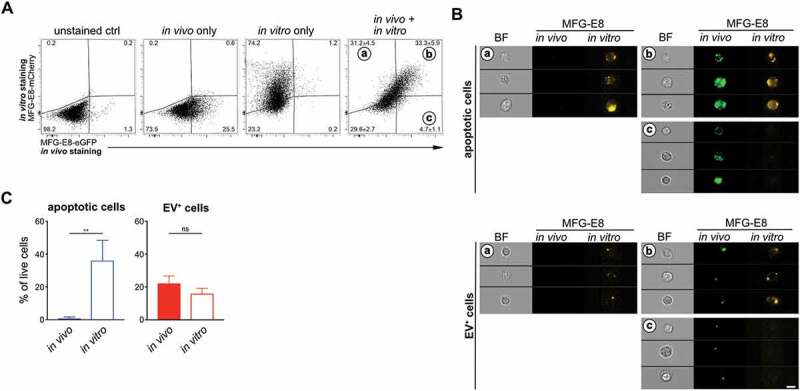
Comparison of *in vivo* and *in vitro* MFG-E8 staining. LCMV_Arm_-infected mice received 100 µg MFG-E8-eGFP iv on day 5 after infection (n = 3). 1h later mice were sacrificed and their spleens homogenised by meshing them through nylon meshes and erythrocytes lysed using standard ACK lysis buffer. Single cell suspensions were further stained with MFG-E8-mCherry followed by imaging flow cytometry analysis. (a) Dot plots show gating of MFG-E8 double-negative, double-positive (gate B), mCherry single positive (gate A) and eGFP single positive (gate C) populations using appropriate unstained and single-stained controls. Double positive cells are cells that were both stained with MFG-E8 *in vivo* and *in vitro*, mCherry single positive cells were only stained *in vitro*, while eGFP single positive cells were only stained *in vivo*. Bar graphs show the result of the CAE analysis that was performed either on MFG-E8-eGFP^+^ *(in vivo* stained) cells (filled bars) or on MFG-E8-mCherry^+^ (*in vitro* stained) cells (open bars). Means ± SDs are shown, apoptotic cells are shown in blue, EV^+^ cells are shown in red. (b) Representative BF, eGFP and mCherry images of MFG-E8-mCherry^+^MFG-E8-eGFP^−^ (gate A), MFG-E8-mCherry^+^MFG-E8-eGFP^+^ (gate B), MFG-E8-mCherry^−^MFG-E8-eGFP^+^ (gate C) are shown. Scale bar 7 µm. Statistical significance is indicated by asterisks (ns *P > 0.05;* **P ≤ 0.01; two-tailed unpaired t-test).

For our analyses we chose *in vivo* stainings of PS^+^ cells, because we were concerned that the organ preparation not only could cause cell death, but also could generate PS^+^ membrane fragments that could attach to cells and render them EV^+^. However, *in vivo* staining is more elaborate and expensive due to the relatively high amounts of recombinant MFG-E8-eGFP required. Therefore, we next assessed whether *in vitro* staining of PS^+^ cells with MFG-E8-eGFP would produce similar results in LCMV-infected mice as the *in vivo* staining. For this, we injected LCMV_Arm_-infected mice with MFG-E8-eGFP prior to their sacrifice to detect the PS^+^ structures which were present *in vivo*. We then prepared single cell suspensions and lysed erythrocytes using ammonium chloride (ACK). The cells were then stained again *in vitro* using MFG-E8-mCherry to differentiate those PS^+^ structures that might have been generated during the tissue preparation. Approx. 30% of all live cells stained double positive for both, MFG-E8-eGFP and MFG-E8-mCherry (Figure 7a). While only few cells (approx. 5%) were stained only *in vivo*, more than 30% were stained only *in vitro* (Figure 7a). This shows that after organ removal and preparation many cells become PS^+^ and that consequently, the *in vitro* staining detected many more PS^+^ cells than the *in vivo* staining (Figure 7a). To assess whether these cells had become apoptotic or bound EVs during the organ preparation, we applied the CAE analysis twice on the same samples, first on *in vivo* stained cells using the eGFP images and then on *in vitro* stained cells using the mCherry images (Figure 7b,c). This analysis revealed that the frequency of EV^+^ cells did not increase, apoptotic cells however, dramatically increased approx. 40-fold (Figure 7c). These results show the importance of the *in vivo* labelling approach, since the organ preparation leads to strong PS^+^ exposure on 40% of all live cells so that they are classified as apoptotic by the CAE analysis.

### EVs carry markers of exosomes and antigen-presenting cells

**Figure 8.**
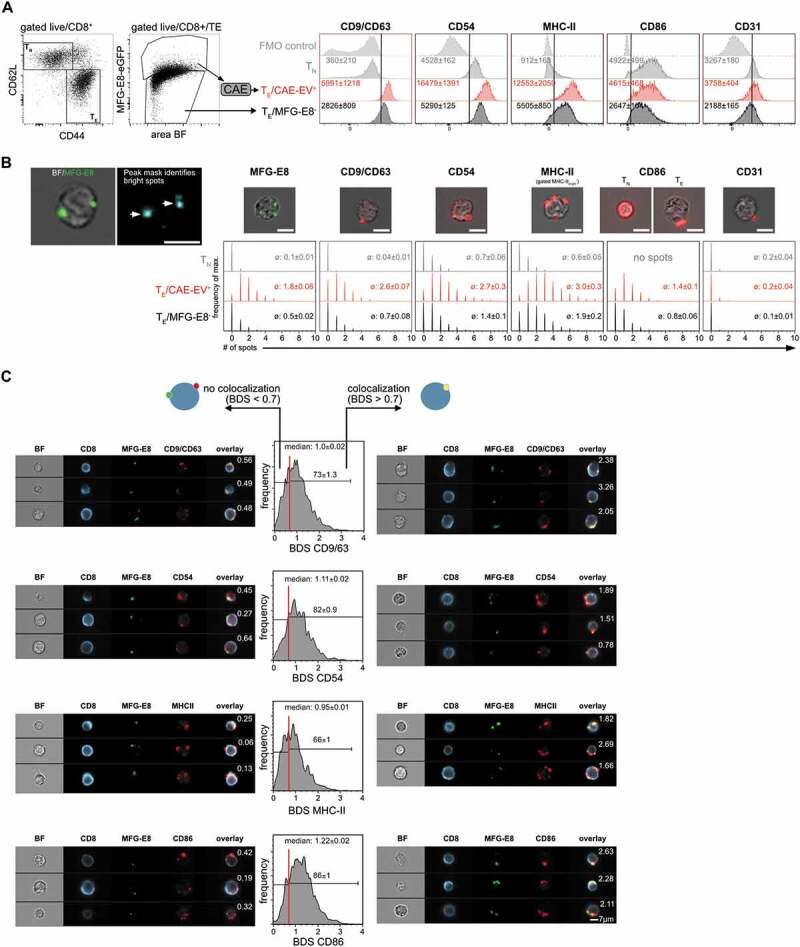
EVs bound to CD8^+^ T cells originate from APCs. (a) Splenic T_N_ and T_E_ CD8^+^ T cells of LCMV-infected mice (day 5 post infection, n = 3) were analysed for staining with MFG-E8 and for expression of potential EV-markers CD9/CD63 (combined in one staining), MHC-II, CD54, CD86 and CD31. Median fluorescence intensities (MFI) ± SD of these proteins are indicated in the histograms. (B) Spot Analysis: Histograms show the number of identified spots on T_N_, EV^+^ T_E_ and MFG-E8^−^ T_E_ CD8^+^ cells. The average number of spots are shown in each histogram. (C) Cells with a BDS < 0.7 did not show any significant co-localisation as determined by visual inspection. Cells with a BDS > 0.7 showed substantial colocalisation of MFG-E8 and the respective EV-marker. BDS scores are shown in the representative example images. Histograms show the BDS scores of EV^+^ T_E_ CD8^+^ cells. The median BDS score and the percentage of cells showing co-localisation (BDS > 0.7) are indicated within histograms. Representative results of two independent experiments are shown.

We next set out to further characterise cell-attached EVs generated during LCMV-infection *in vivo* using the flow microscopy CAE pipeline. EVs originating from APCs like DCs or B cells carry exosomal markers such as tetraspanins CD9 and CD63 related to endosomal vesicle trafficking [55,56]. In addition, they are enriched for APC-markers such as CD86, MHC-II and CD54 [57]. We therefore evaluated next if EV^+^ T cells are positive for these APC-exosome markers. Analysis of naïve CD44^−^CD62L^+^ T_N_ cells and effector CD44^+^CD62L^−^ T_E_ cells from the same LCMV-infected animal showed that EVs bound specifically to activated CD44^+^ T_E_ cells, but not to resting CD44^−^ T_N_ cells (Figure 6c, Suppl. Fig. 5A,B). We first compared the MFIs of CD9/CD63 (combined in one staining), CD54, MHC-II, CD86 and CD31 between CD8^+^ T_N_, MFG-E8^−^CD8^+^ T_E_ and EV^+^CD8^+^ T_E_ (Figure 8a). We found strongly increased MFIs of CD9/CD63, CD54 and MHC-II on EV^+^CD8^+^ T_E_ cells as compared to MFG-E8^−^ CD8^+^ T_E_ or CD8^+^ T_N_ (Figure 8a), which were statistically significantly elevated on EV^+^CD8^+^ T_E_ cells for CD9/CD63, CD54, MHC-II (Suppl. Fig. 7A). As T cells of the mouse do not express MHC-II themselves [58], this supports the possibility that T_E_ acquire these molecules through EVs.

Indeed, all markers showed a spot-like staining pattern on T cells, similar to MFG-E8^+^ EVs (Figure 8b). An exception was CD86, where CD8^+^ T_N_ showed a complete surface staining, while its staining pattern on CD8^+^ T_E_ changed to a spot-like appearance (Figure 8b). The MFI of CD86 was higher on CD8^+^ T_N_ cells, as they express this molecule themselves and downregulate CD86 in the CD44^+^ memory state, as published previously [59]. Yet, EV^+^ T_E_ cells had a higher CD86 MFI than MFG-E8^−^ T_E_ (Figure 8a Suppl. Fig 7A) suggesting accumulation of CD86 via EV-association to T_E_ cells. These observations were not only restricted to CD8^+^ T cells, but were also true for CD4^+^ T cells (Supplemental Fig. 8A,B). To further investigate whether T_E_ cells acquired the marker molecules through EVs, we generated a spot mask in the IDEAS software to quantify the number of MFG-E8^+^ and EV-marker^+^ spots. The spot count was significantly increased for all analysed markers on EV^+^ T_E_ compared to MFG-E8^−^ T_E_ or T_N_ of the CD8^+^ (Figure 8b, Suppl. Fig. 7B) or CD4^+^ (Suppl. Fig. 8B) T cell compartment, further supporting the idea that CD8^+^ and CD4^+^ T_E_ cells acquire these molecules through EVs. If this was indeed the case, substantial colocalisation between MFG-E8 and the EV-markers should occur. For quantifying the degree of colocalisation we used the bright detail similarity (BDS) feature on the peak masks in the IDEAS software. When a cells’ BDS score was >0.7, we found substantial colocalisation between the MFG-E8 and the EV-marker staining. This analysis revealed that approximately 73% of all EV^+^ CD8^+^ T_E_ showed high colocalisation of MFG-E8 with CD9/CD63^+^, 82% with CD54, 66% with MHC-II and 86% with CD86 (Figure 8c). CD4^+^ EV^+^ T_E_ showed similar results (Suppl. Fig. 8 C). As mouse T cells do not express MHC-II, neither as naïve, nor as activated cells, these molecules found on the surface of T cells and colocalising with MFG-E8-eGFP must be of APC-origin. Furthermore, CD44^+^CD8^+^ T_E_ cells do not express CD86 on their surface [59] and CD86 colocalises to a great part with MFG-E8, the spot-like CD86 staining in T_E_ most likely is the result of binding of CD86^+^PS^+^ EVs to T cells. There was also a certain percentage of CD8^+^ (Figure 8c) and CD4^+^ (Suppl. Fig. 8 C) T cells with a BDS score <0.7 detected. Here we found EVs where MFG-E8 did not colocalise with any of the above markers. This argues either for the existence of PS ^–^ EVs where MFG-E8 staining was absent or below the detection limit or other processes of protein transfer to activated T cells such as trogocytosis [60]. However, these EVs were in the minority.

The endothelial cell marker CD31 was included in the analysis as potential marker for apoptotic bodies, since many CD31^+^ endothelial cells die by apoptosis early during LCMV infection [61]. We only found weak staining for CD31 on CD8^+^ T cells (Figure 8a), and hardly any CD31^+^ spots (Figure 8b). To the contrary, CD4^+^ T cells showed CD31 surface expression (Suppl. Fig. 8A), but also no CD31^+^ spots. These results indicate that most EVs detected on activated T cells are not apoptotic bodies, but rather exosomes that are derived from APCs.

## Discussion

Here we report the *in vivo* application of recombinant MFG-E8-eGFP for the detection of PS^+^ cells using conventional and image flow cytometry. We show that MFG-E8-eGFP binds similar fractions of PS^+^ apoptotic cells *in vitro* as compared to the more widely used Annexin V. However, PS-binding of Mge8-eGFP is calcium independent and works after *in vivo* injection. Surprisingly, we found the majority of PS^+^ cells in irradiated and LCMV-infected mice being not apoptotic, but alive and decorated with PS^+^ EVs. Upon development of a deep learning autoencoder, we can faithfully separate both, true apoptotic cells and EV-decorated cells for further analyses.

The detection of cell death in tissues *in vivo* is challenging. Therefore, dead cell analyses are mostly performed *ex vivo* with single cell suspensions derived from the organs of interest after biopsies or sacrifice of experimental animals. However, this procedure exposes cells to additional stress factors such as shear forces by tissue homogenisation, enzymes, temperature and pH changes, salt compositions of working solutions and many more. We can demonstrate that tissue preparation for dead cell analysis *ex vivo* artificially increases cell death rates due to handling. In addition, most methods to measure apoptotic cells have intrinsic restrictions, adding to their imprecision of analysing cell death. For example, labelling of fragmented DNA by TUNEL (terminal deoxynucleotidyl transferase dUTP nick end labelling) mostly detects late stage apoptotic cells only [62], which are very rapidly cleared *in situ*, as most DNA fragmentation of dying cells occurs inside phagocytes [63]. Using the analysis pipeline presented in this report, we could detect many phagocytes attached to apoptotic cells, probably for engulfment and efferocytosis [3]. Furthermore, measuring the active form of caspase-3 using fluorescent substrates is not completely specific, as caspase-3 is also activated independently of cell death in certain cell types [64]. The *ex vivo* staining of cells for surface PS using Annexin V has the disadvantage to require high Ca^2+^-levels, precluding it from most *in vivo* applications and interfering with many other downstream applications [18]. MFG-E8, also known as lactadherin, also binds to PS on apoptotic cells [19]. However, translocation of PS to the outer membrane not only occurs during apoptosis, but also during the formation of microvesicles [6,8] and exosomes [9], allowing detection of PS^+^ EVs by MFG-E8-eGFP.

Although we could detect many MFG-E8^+^ cells, true apoptotic cells were relatively rare in spleens and BM of control mice, while the great majority were EV-decorated live cells. Due to their great morphological variability, a reliable discrimination was not possible using fluorescent intensity of the MFG-E8 signal only. Also, a combination of two features that worked well on manually selected apoptotic and EV-decorated cells failed in more complex samples. Only *in vivo* administered MFG-E8-eGFP in combination with imaging flow cytometry and a deep learning approach using a CAE-RF allowed us to reliably classify the MFG-E8^+^ cells into PS^+^ apoptotic or PS^+^ EV-decorated cells. Using this pipeline, we detected significant cell death during LCMV-infection specifically among CD4^+^ and CD8^+^ T_CM_ cells. Previous reports showed substantial death among CD44^+^CD4^+^ and CD8^+^ memory T cells during the early phase (day 2–4) of LCMV infection. Such attrition depended on type I interferon production [52–54]. Our novel method, using a single staining reagent, therefore confirmed previous findings obtained with other, more laborious methods involving combinations of *in vitro* culture, Annexin V-staining, TUNEL-assays and active Caspase detection [52–54].

Another striking finding is the dramatic increase in cells that bind EVs. On the one hand these vesicles could be virus-containing particles infecting new cells, or apoptotic bodies reflecting the increased amount of cell death. However, given the wide range of immunoregulatory functions of EVs [14], the increase in EVs could also be a consequence of the ongoing immune response. The fact that especially B cells and activated CD8^+^ T cells bind these EVs, supports this idea. It has been shown previously that activated DCs secrete MHC-II containing exosomes, which bind to activated CD4^+^ T cells via LFA-1 [65] and could play a role in T cell activation [55]. Evidence for their strong immunostimulatory function came from early exosome studies demonstrating the capability of tumour-antigen bearing exosomes secreted from DCs to trigger T-cell dependent anti-tumour responses [56]. In LCMV-infected mice CD4^+^ T cells were not as strongly EV-decorated as compared to CD8^+^ T cells. Previous reports showed that vesicles derived from DCs were able to stimulate CD8 T cells *in vitro* [66] and transfer exogenous antigen to DCs for CD8^+^ [67] and CD4^+^ T cell priming [68].

Approximately half of all CD8^+^ T cells were EV-decorated during LCMV infection. This could indicate that either those T cells were targeted by EVs produced by other cells, or that we detected nascent EVs produced by T cells themselves. Both scenarios are possible. It has been shown that TNFα-containing exosomes were able to delay activation-induced cell death in T cells [69]. On the other hand, T cells release EVs constitutively and EV secretion is enhanced by TCR triggering [70,71], which causes increased intracellular calcium levels for enhanced EV-production [72]. Moreover, EVs from CD8^+^ T cells may also contain granzyme and perforin [73] and can inhibit antigen presentation and survival of DCs to downmodulate immune responses in mouse models of cancer and diabetes [74]. In addition, microvesicles budding from the immunological synapses of CD4^+^ T cells *in vitro* do contain TCR, which may transfer signals to B cells expressing cognate peptide MHC-II [75]. However, our finding that MFG-E8^+^ EVs carry APC-markers such as MHC-II, CD86, CD54 and tetraspanins rather argue for the exosomal APC-origin of these EVs. Especially the fact that MHC-II is not made by murine CD4^+^ or CD8^+^ T cells strongly argues for the fact that these EVs are APC-derived.

Also, many B cells carried vesicles, even in non-infected mice. EVs can be a source of native, unprocessed antigen [76] and in the case of virus infections they could carry intact viral proteins [77] for recognition by cognate B-cell receptors causing B cell activation. In addition, previous studies described B cells becoming Annexin V positive, without undergoing apoptosis [78]. While the authors described that PS-exposure was selective for B cells upon their IgM-mediated positive selection, they did not determine, if PS-exposure was cell-intrinsic or by EV-decoration.

Further studies are necessary to determine if EV binding is restricted to certain zones of lymphatic organs, such as the MZ, where blood is filtered. Cells locating to the MZ, such as MZ B cells, CD11 c^+^CD11b^−^ DCs and activated T cells could therefore be preferentially exposed to EVs. However, the fact that also follicular B cells are EV-decorated argues against this possibility.

To our knowledge, this is the first report that identifies cell subsets binding naturally occurring EVs *in vivo. In vivo* staining of dying cells and EV-decorated cells using MFG-E8-eGFP is a valuable tool, not only to reliably identify cells that undergo cell death in different pathological conditions, but also to clarify the function of EVs on different cell types in various tissues under normal and pathological conditions, such as viral infections, autoimmunity and cancer.

## Supplementary Material

Supplemental Material
