# Supplementary material for: In vivo identification of apoptotic and extracellular vesicle-bound live cells using image-based deep learning
Source: J Extracell Vesicles. 2020 Jul 16;9(1):1792683. doi: 10.1080/20013078.2020.1792683 (PMC7480589; doi:10.1080/20013078.2020.1792683)
Supplement: Supplemental Material [file ZJEV_A_1792683_SM6653.docx]

**Supplemental Information (Kranich et al.)**

**
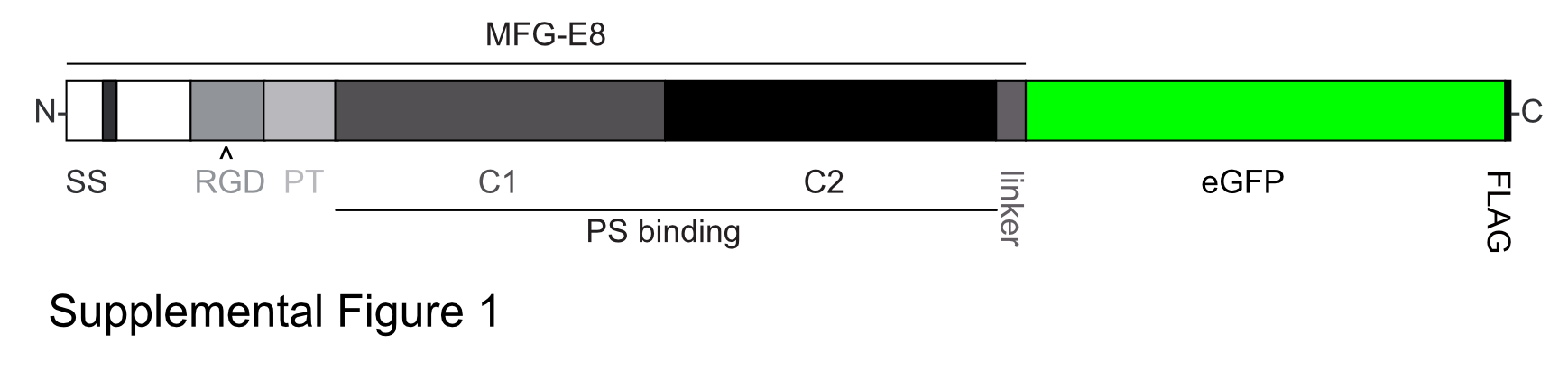
**

**Supplemental Figure 1: MFG-E8-eGFP fusion protein**

Enhanced green fluorescent protein (eGFP) was fused to the C-terminus of full-length murine MFG-E8, separated by a 15aa helical linker (linker). To aid purification, a FLAG-tag was added to the C-terminus of eGFP. PT, prolin-threonine rich domain; RGD, RDG-motif; SS, signal sequence; N, N-terminus; C, C-terminus; C1, C1-domain; C2, C2-domain; PS, phosphatidylserine.

**
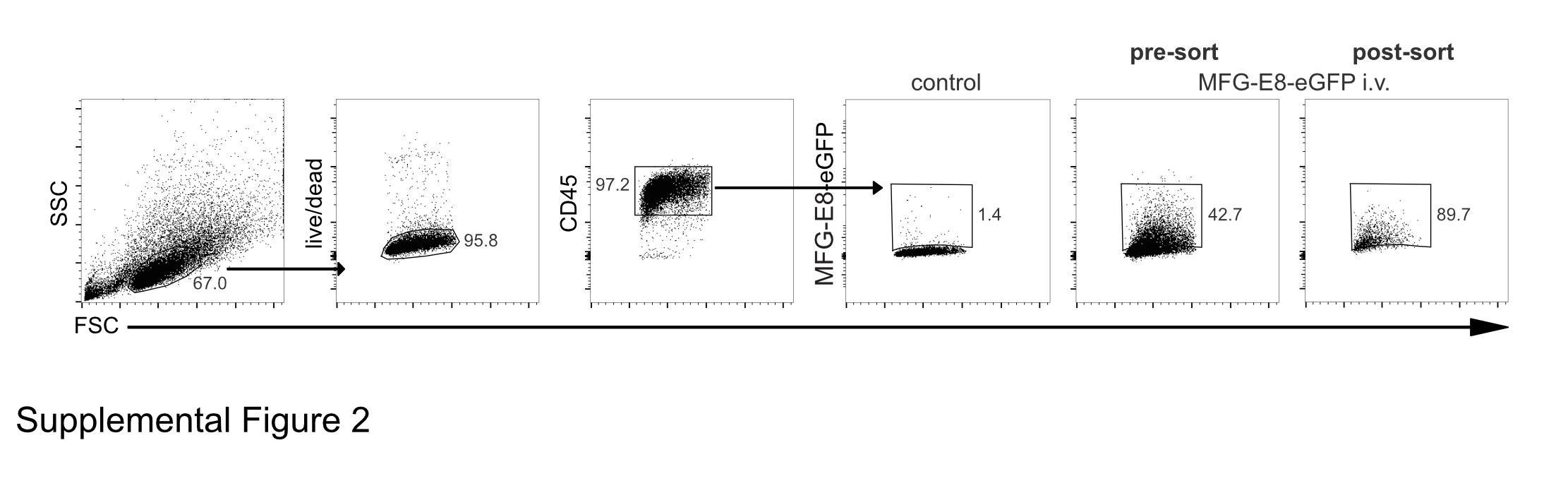
**

**Supplemental Figure 2: Sorting strategy for MFG-E8^+^ splenocytes**

LCMV_Arm_ infected mice (day 5 post infection) were injected with 100µg MFG-E8-eGFP i.v. 1h prior to sacrifice. Live/dead^-^CD45^+^MFG-E8-eGFP^+^ splenocytes were sorted. Sort purity of MFG-E8-eGFP^+^ cells was approx. 90%. Cells were then analyzed for the presence of extracellular vesicles by TEM.

**
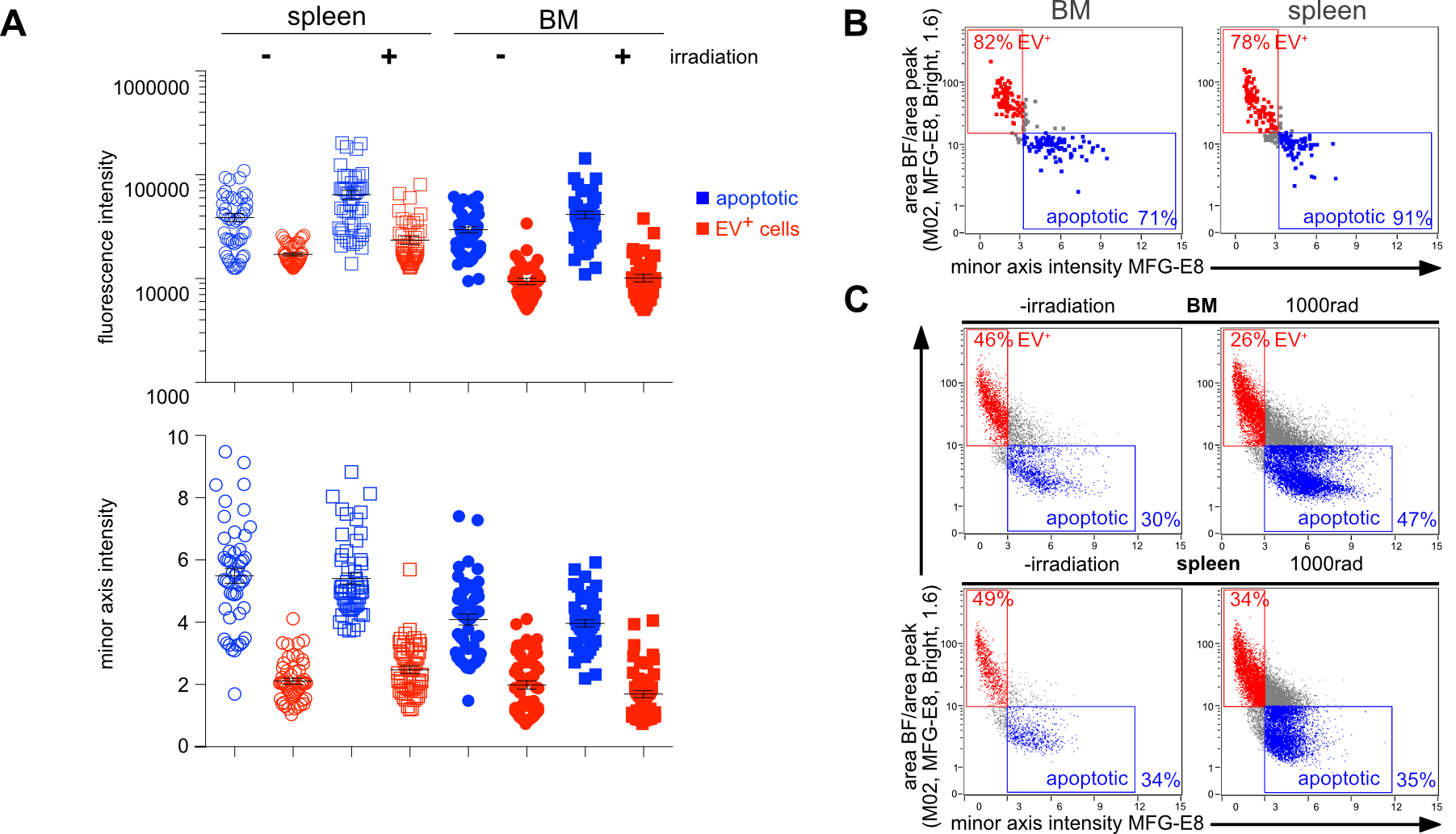
**

**Supplemental Figure 3: Comparison of MFG-E8-eGFP MFI and minor axis intensity and IDEAS gating strategy.**

(A) Mean MFG-E8-eGFP fluorescence intensities (top) and MFG-E8-eGFP minor axis intensities (bottom) of 200 manually selected apoptotic cells and 200 EV^+^ cells from irradiated and non-irradiated BM cells and splenocytes are shown. Using minor axis intensities resulted in a good separation of apoptotic and EV^+^ cells in both spleen and BM, while using the MFI of MFG-E8-eGFP did not result in a good separation of apoptotic and EV^+^ cells in BM. (B) 200 apoptotic (blue) and 200 EV^+^ cells (red) were manually selected from non-irradiated and irradiated spleen and BM samples. Dot plots show their MFG-E8-eGFP minor axis intensity values and the ratio of the BF area and the area of the MFG-E8-eGFP signal. Apoptotic cells were defined as having a MFG-E8-eGFP minor axis intensity value >3 and an area ratio <10, EV^+^ cells as having a MFG-E8-eGFP minor axis intensity <3 and an area ratio >10. Percentages show the frequency of manually selected cells that were correctly classified by the IDEAS features. (C) Apoptotic cells (blue) and EV^+^ cells (red) from total spleen and BM from irradiated and non-irradiated mice were quantified using the BF:MFG-E8-eGFP area ratio and the MFG-E8-eGFP minor axis intensity. Uncategorized cells are shown in gray.

**Supplemental Figure 4: Flow cytometry gating strategies.**

(A) General gating strategy for MFG-E8^+^ apoptotic and EV^+^ cells is shown. First, single cells are gated using the aspect ratio and the area of the BF signal. Then necrotic cells are removed by gating live/dead viability dye negative cells (live). If required, autofluorescent cells were removed by using an empty channel (channel 4). Then MFG-E8-eGFP^+^ cells were gated using a negative control (PBS injection). MFG-E8-eGFP^+^ cells were sorted into apoptotic and EV^+^ cells using a CAE. (B) Gating strategy for different B cell subsets: First, all B cells were gated using CD19. Then different subsets were gated based on their CD21 and CD23 expression (MZ = marginal zone, CD21^+^CD23^-^; FO = follicular, CD21^+^CD23^+^; IM = immature B cells, CD21^-^CD23^-^). (C) Gating strategy to analyze DCs: First, non-lymphocytes were gated as CD19^-^/TCRβ^-^ cells. Then DCs were gated as CD11c^+^MHCII^+^.

**
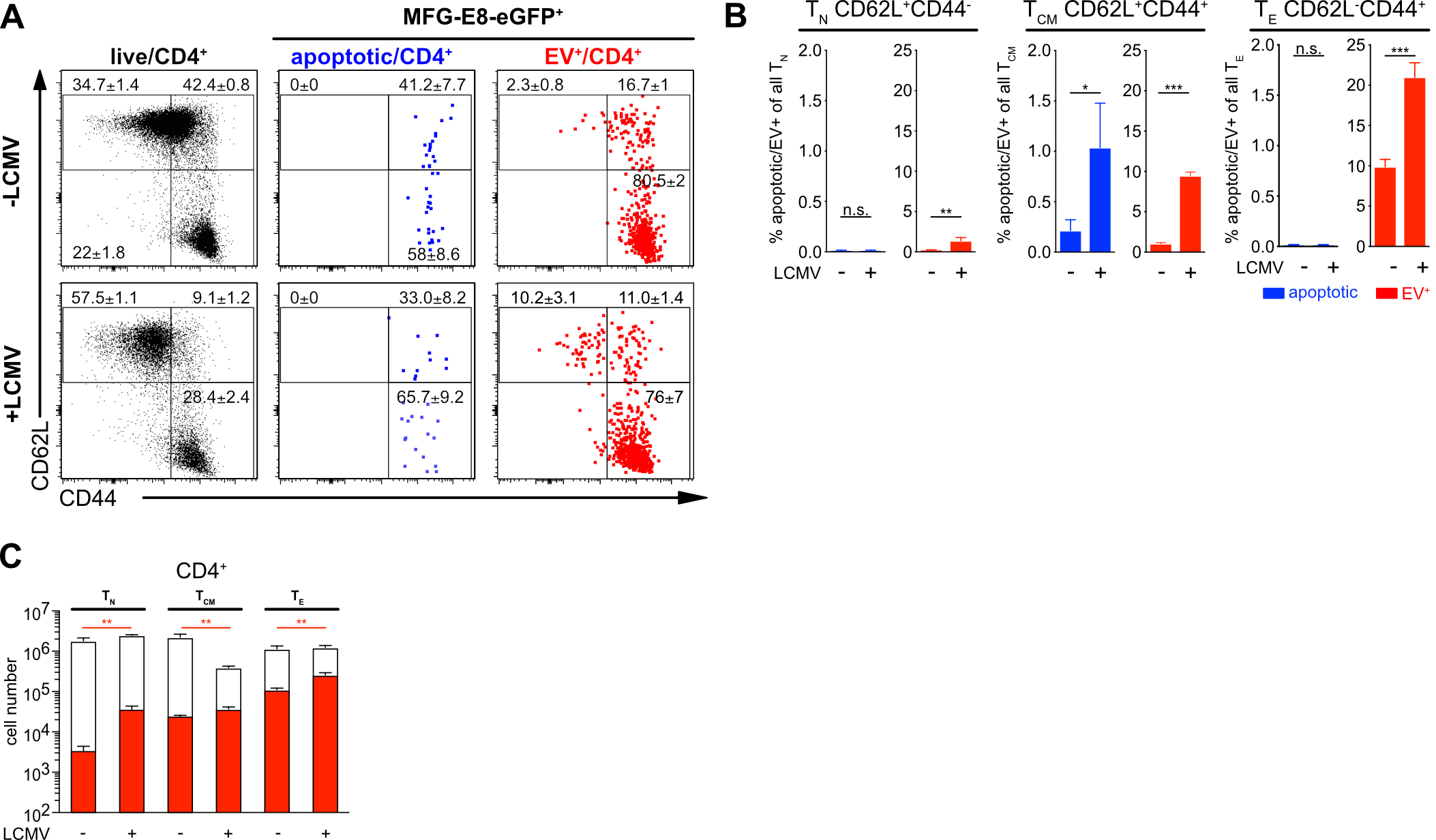
**

**Supplemental Figure 5: Activated CD4^+^ T cells bind EVs during LCMV infection.**

(A) CD4^+^ T cells were further subdivided into T_N_ (CD62L^+^CD44^-^), T_CM_ (CD62L^+^CD44^+)^ and T_E_ (CD62L^-^CD44^+^) cells. Using the CAE their frequencies of apoptotic and EV^+^ cells were analyzed. (B) Bar graphs show the average frequencies of MFG-E8-eGFP^+^ apoptotic (blue) and EV^+^ (red) CD8^+^ T_N_, T_CM_ and T_E_ cells. (C) Total numbers of CD8^+^ T_N_, T_CM_ and T_E_ cells were determined (white bar) and plotted against the total numbers of EV^+^ subsets (red bar). For statistical analysis Student’s T test was used. Statistical significance is indicated by asterisk (ns *P>0.05;* **P*≤0.05; ***P*≤0.01; ****P*≤0.001; two-tailed unpaired t-test). Representative results of 3 independent experiments are shown.

**Supplemental Figure 6: RGD-motif is dispensable for binding of EVs to activated CD8^+^ T cells.**

(A) Enhanced green fluorescent protein (eGFP) was fused to the C-terminus of MFG-E8 lacking the E1, E2 and PT domain (C1C2), separated by a 15aa helical linker, followed by a FLAG -tag. SS, signal sequence; N, N-terminus; C, C-terminus; C1, C1-domain; C2, C2-domain; PS, phosphatidylserine. (B) LCMV_Arm_ (2x10^5^ PFU, i.p.) infected mice were injected with 100µg C1C2-eGFP (n=3) or 80µg MFG-E8-eGFP (n=3) on day 5 post infection. 1h later mice were sacrificed and eGFP^+^ cells analyzed by CAE. Dot plots show gating of eGFP^+^ live cells. Blue bar graphs show % of apoptotic cells of eGFP^+^ cells, red bar graphs show % of EV^+^ cells of eGFP^+^ cells. Closed and open bars show cells stained with full-length MFG-E8-eGFP and C1C2-eGFP, respectively. (C) Dot plots show CD62L and CD44 expression of all live CD8^+^ (black) or EV^+^CD8^+^ T cells (red) stained either with MFG-E8-eGFP (upper panel) or C1C2-eGFP (lower panel). Numbers next to the gate show the mean percentage ± SD of all cells depicted within the dot plot that lie within the respective gate. Bar graphs show % EV^+^CD8^+^ T_E_ of all eGFP^+^ cells stained either with full-length MFG-E8 (closed bars) or C1C2 (open bars). For statistical analysis Student’s T test was used (n=3). Statistical significance is indicated (ns *P>0.05;* two-tailed unpaired t-test.).

**
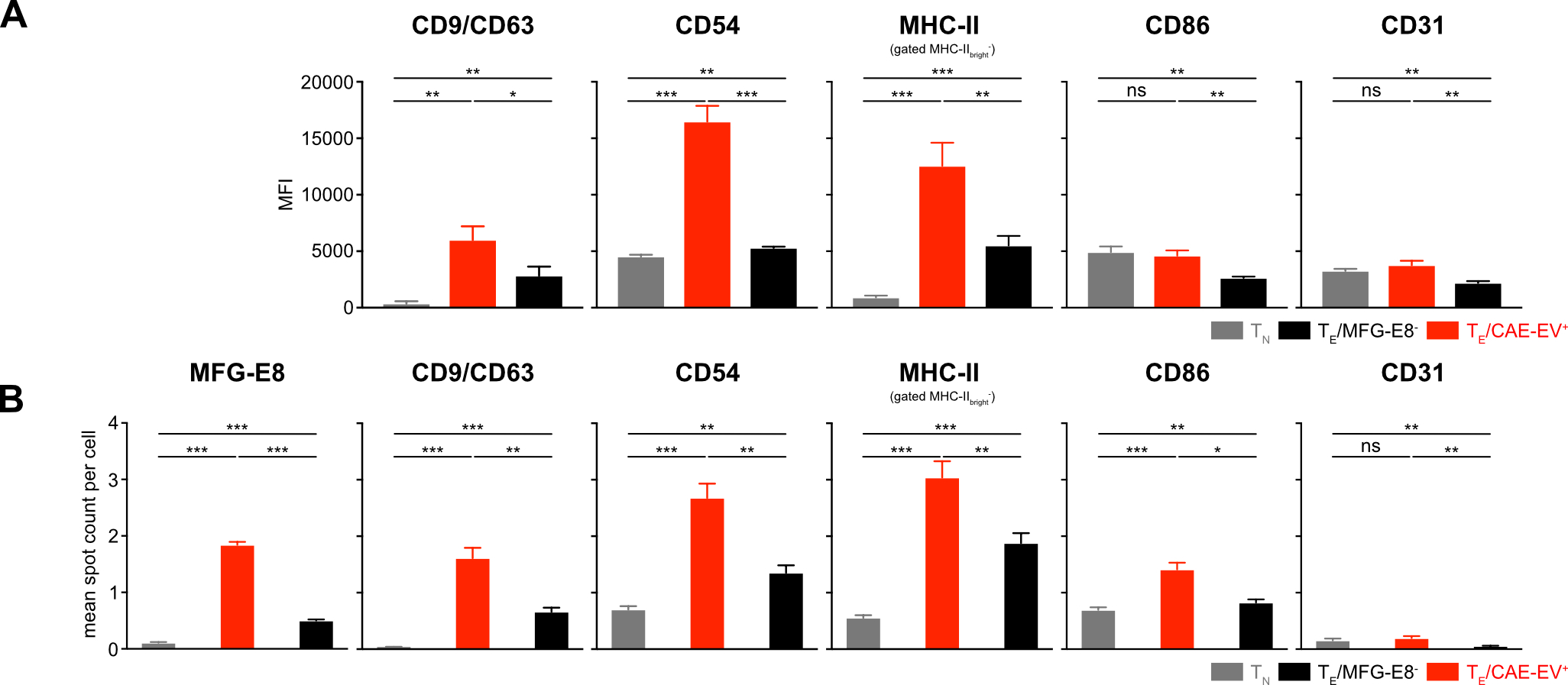
**

**Supplemental Figure 7: Statistical analysis of MFI and spot count of EV-markers in CD8^+^ T cells.**

Statistical analysis of EV-marker analysis of CD8^+^ T cells as shown in Figure 8. (A) Bar graphs show average MFIs ± SD in T_N_ (gray), EV^+^ T_E_ (red) and MFG-E8^-^ T_E_ (back). (B) Bar graphs show average spot counts ± SD in T_N_ (gray), EV^+^ T_E_ (red) and MFG-E8^-^ T_E_ (back). For statistical analysis Student’s T test was used (n=3). Statistical significance is indicated by asterisk (ns *P>0.05;* **P*≤0.05; ***P*≤0.01; ****P*≤0.001; two-tailed unpaired t-test). Representative results of 2 independent experiments are shown.

**
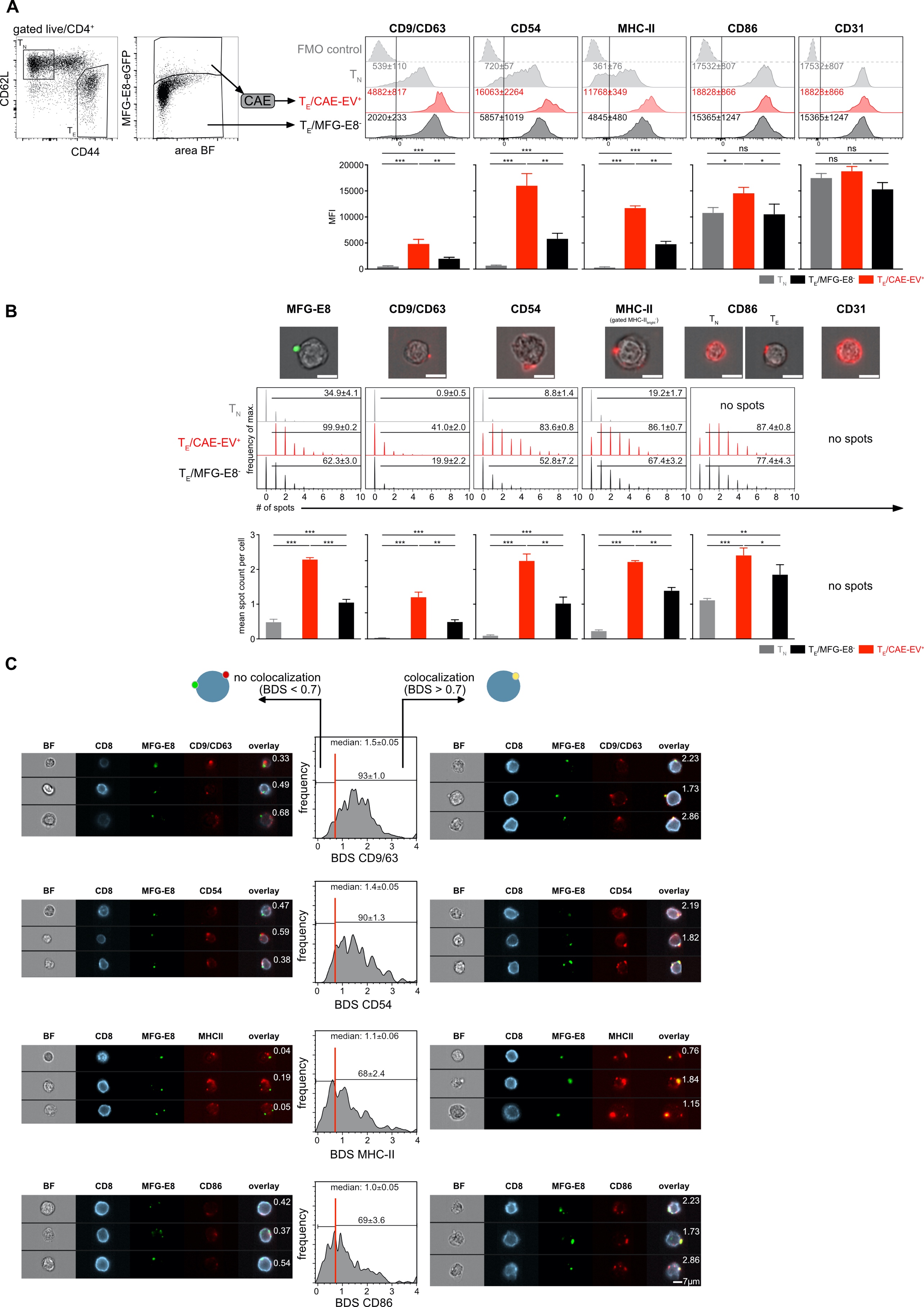
**

**Supplemental Figure 8. EVs bound to CD8+ T cells originate from APCs.**

(A) Splenic TN and TE CD4+ T cells of LCMV-infected mice (day 5 post infection, n=3) were analyzed for staining with MFG-E8 and for expression of potential EV-markers CD9/CD63 (combined in one staining), MHCII, CD54, CD86 and CD31. Median fluorescence intensities (MFI) ± SD of these proteins are indicated in the histograms. Bar graphs show average MFIs ± SD in TN (gray), EV+ TE (red) and MFG-E8- TE (back). (B) Spot analysis: The spot count feature of the IDEAS mask was used to quantify the number of spots. Imaging flow cytometry images of BF and MFG-E8 or BF and the EV-markers show the spot-like staining pattern of these markers. Histograms show the number of identified spots on TN, EV+ TE and MFG-E8- TE CD8+ cells. Frequencies of spot+ cells and the average number of spots are shown in each histogram. Bar graphs show average spot counts ± SD in TN (gray), EV+ TE (red) and MFG-E8- TE (back). (C) To determine if EV-marker+ and MFG-E8+ spots colocalized the bright detail similarity (BDS) feature of the IDEAS software. Cells with a BDS < 0.7 did not show any significant co-localization as determined by visual inspection. Cells with a BDS > 0.7 showed substantial co-localization of MFG-E8 and the respective EV-marker. BDS scores are shown in the representative example images. Histogram show the BDS scores of EV+ TE CD8+ cells. The median BDS score and the percentage of cells showing co-localization (a BDS > 0.7) are indicated within histograms (n=3). Statistical significance is indicated by asterisk (**P*≤0.05; ***P*≤0.01; ****P*≤0.001; two-tailed unpaired t-test).

**Suppl. Table 1: Staining reagents and antibodies used in this study.**

| **anti-mouse antibodies** |  |  |  |  |
| --- | --- | --- | --- | --- |
| **reactivity/staining reagent** | **conjugate** | **clone** | **Cat. #** | **vendor** |
| CD11b | APC | M1/0 | 17-0112-82 | eBioscience |
| CD11c | PE/Cy7 | N418 | 117318 | BioLegend |
| CD127 | PE | (SB/199) | 121112 | BD Biosciences Pharmingen |
| CD19 | APC/eFluor780 | eBio1D3 | 47-0193-82 | eBioscience |
| CD19 | PE/Cy7 | 6D5 | 115520 | BioLegend |
| CD19 | APC | 1D3 | 152410 | BD Biosciences Pharmingen |
| CD21/35 | APC/Cy7 | 7E9 | 123418 | BioLegend |
| CD23 | PE/Cy7 | B3B4 | 101614 | BioLegend |
| CD28 | purified | 37,51 | BE0015-1 | BioXCell |
| CD3 | purified | 145-2C11 | BE0001-1 | BioXCell |
| CD31 | AF647 | MEC13.3 | 102516 | BioLegend |
| CD4 | APC | RM4-5 | 100516 | BioLegend |
| CD44 | APC/Cy7 | IM7 | 103028 | BioLegend |
| CD44 | Pacific Blue | IM7 | 103028 | BioLegend |
| CD45 | AF647 | 30-F11 | 103124 | BioLegend |
| CD45.1 | BV421 | A20 | 10731 | Biolegend |
| CD51 | PE | RMV-7 | 104106 | Biolegend |
| CD54 | AF647 | YN1/1.74 | 116114 | Biolegend |
| CD61 | PE | 2C9.G2 | 104308 | Biolegend |
| CD62L | PE | MEL-14 | 12-0621-82 | eBioscience |
| CD63 | APC | NVG-2 | 143905 | eBioscience |
| CD8a | PE/Cy7 | 53-6.7 | 100722 | BioLegend |
| CD86 | PE | GL1 | 12-0862-82 | eBioscience |
| CD9 | APC | eBioKMC8 | 17-0091-82 | eBioscience |
| CD90.1 | APC/Fire750 | X-7 | 202543 | BioLegend |
| cleaved Cas8 | purified | D5B2 | 8592S | Cell Signaling |
| GFP | FITC | polyclonal | ab662 | Abcam |
| KLRG-1 | PE/Cy7 | 2F1 | 25-5893-82 | eBioscience |
| MHC-II (I-A/I-E) | PE/Cy7 | M5/114.15.2 | 25-5321-82 | eBioscience |
| NFATc1 | PE | 7A6 | 2649605 | Biolegend |
| TCRbeta | APC/Cy7 | H57-597 | 109219 | BioLegend |
|  |  |  |  |  |
| **secondary antibodies** |  |  |  |  |
| **reactivity/ staining reagent** | **conjugate** | **clone** | **Cat. #** | **vendor** |
| goat anti-rabbit IgG (H+L) | AF647 | polyclonal | A32733 | Invitrogen |
|  |  |  |  |  |
| **other staining reagents** |  |  |  |  |
| **reactivity/ staining reagent** | **conjugate** |  | **Cat. #** | **vendor** |
| AnnexinV | Cy5 |  | ab14150 | Abcam |
| Draq5 |  |  | 424101 | BioLegend |
| live/dead violet |  |  | L34955 | Thermo Fisher |
| PKH26 |  |  | PKH26GL-1KT | Sigma Aldrich |
